# The High Light Response in Arabidopsis Requires the Calcium Sensor Protein CAS, a Target of STN7- and STN8-Mediated Phosphorylation

**DOI:** 10.3389/fpls.2019.00974

**Published:** 2019-07-30

**Authors:** Edoardo Cutolo, Nargis Parvin, Henning Ruge, Niloufar Pirayesh, Valentin Roustan, Wolfram Weckwerth, Markus Teige, Michele Grieco, Veronique Larosa, Ute C. Vothknecht

**Affiliations:** ^1^Plant Cell Biology, Institut für Zelluläre und Molekulare Botanik, University of Bonn, Bonn, Germany; ^2^Department of Plant Nutrition, Institut für Nutzpflanzenwissenschaften und Ressourcenschutz, University of Bonn, Bonn, Germany; ^3^Department of Biology I, Ludwig Maximilian University of Munich, Munich, Germany; ^4^Department of Molecular Systems Biology, University of Vienna, Vienna, Austria; ^5^Leibniz Institute of Plant Genetics and Crop Plant Research (IPK), Gatersleben, Germany; ^6^Laboratory of Genetics and Physiology of Microalgae, InBios, University of Liège, Liège, Belgium

**Keywords:** calcium sensor protein, CAS, state transition, STN7, STN8, phosphorylation, chloroplast, photoacclimation

## Abstract

Reversible phosphorylation of thylakoid proteins contributes to photoacclimation responses in photosynthetic organisms, enabling the fine-tuning of light harvesting under changing light conditions and promoting the onset of photoprotective processes. However, the precise functional role of many of the described phosphorylation events on thylakoid proteins remains elusive. The calcium sensor receptor protein (CAS) has previously been indicated as one of the targets of the state transition kinase 8 (STN8). Here we show that in *Arabidopsis thaliana*, CAS is also phosphorylated by the state transition kinase 7 (STN7), as well as by another, so-far unknown, Ca^2+^-dependent kinase. Phosphoproteomics analysis and *in vitro* phosphorylation assays on CAS variants identified the phylogenetically conserved residues Thr-376, Ser-378, and Thr-380 as the major phosphorylation sites of the STN kinases. Spectroscopic analyses of chlorophyll fluorescence emission at 77K further showed that, while the *cas* mutant is not affected in state transition, it displays a persistent strong excitation of PSI under high light exposure, similar to the phenotype previously observed in other mutants defective in photoacclimation mechanisms. Together with the observation of a strong concomitant phosphorylation of light harvesting complex II (LHCII) and photosynthetic core proteins under high irradiance in the *cas* mutant this suggests a role for CAS in the STN7/STN8/TAP38 network of phosphorylation-mediated photoacclimation processes in Arabidopsis.

## Introduction

In their daily life, plants experience drastic fluctuations of the light environment that could significantly impact their photosynthetic activity. Accordingly, rapid and dynamic adjustment mechanisms and multi-layered control strategies have evolved in phototrophic organisms to ensure proper functionality of the photosynthetic apparatus, especially under limiting or stressing light conditions (Rochaix, 2011). Efficient, light-dependent photosynthetic ATP and NADPH production relies in the first place on the balanced excitation of the two photosystems (PSI and PSII), and on the unimpaired electron flow through the electron transport chain (ETC). Operational resilience of the photosynthetic machinery is achieved through a series of built-in mechanisms that enable a fast acclimation to the changing environment and ensure protection of sensitive photosynthetic core components from damage caused by excessive light (Tikkanen et al., 2010; Pesaresi et al., 2011; Rochaix, 2011; Larosa et al., 2018).

Under limiting light conditions, excitation energy can be balanced between the two photosystems via the so-called phenomenon of state transitions (Bonaventura and Myers, 1969; Murata, 1969). This highly conserved adaptive mechanism (Ruban and Johnson, 2009; Papageorgiou and Govindjee, 2011; Kodru et al., 2015) is mediated by reversible protein phosphorylation and represents an important short-term acclimation mechanism enabling photosynthetic organisms to actively respond to rapid changes in light conditions (Rochaix, 2014). The canonical model of state transitions proposes that a mobile fraction of the light-harvesting complex II (LHCII) antenna is dynamically relocated between PSI and PSII in response to the prevailing light condition. This mechanism transiently modifies the size of the relative antenna cross section of the two photosystems and alters their light harvesting capability, thus promoting optimal photon use and preventing redox imbalances along the electron transfer chain. Reversible phosphorylation of LHCII depends on the antagonistic activities of the thylakoid-localized state transition kinase 7 (STN7) and the thylakoid-associated phosphatase 38 (PPH1/TAP38) (Pesaresi et al., 2010; Pribil et al., 2010; Shapiguzov et al., 2010). While STN7 is activated through sensing of over-reduction of the plastoquinone pool (Vener et al., 1997), PPH1/TAP38 is believed to be constitutively active (Silverstein et al., 1993; Pribil et al., 2010). In this model, STN7 and TAP38 thus regulate the excitation balance between PSI and PSII by phosphorylating/de-phosphorylating mobile LHCII trimers and causing their preferential association with PSI or PSII, which are enriched in different regions of the thylakoid membrane system (Tikkanen et al., 2006; Minagawa, 2011; Tikkanen and Aro, 2012). This model has been nevertheless partially challenged recently by findings that indicate that LHCII is found phosphorylated independently from its localization within the thylakoid membrane structure (Mekala et al., 2015). More research is required to truly understand the intricate network of protein phosphorylation, lateral complex migration and protein interactions that enable the optimization of photosynthetic energy fluxes.

A close paralog of STN7, the state transition kinase 8 (STN8), is mainly responsible for the phosphorylation of a set of PSII core components, including the D1, D2, and CP43 proteins, especially under high irradiance (Bonardi et al., 2005; Vainonen et al., 2005). For this reason, the role of STN8 has been connected with the repair cycle of photodamaged PSII reaction centers, where it appears to regulate the displacement of damaged D1 proteins (Fristedt et al., 2009). However, phosphorylation of PSII core proteins and thus STN8 are also part of the broader array of phosphorylation-dependent photoacclimation mechanisms (Mekala et al., 2015).

Aside from LHCII and the PSII core components, additional phosphoproteins populate the thylakoid membrane system, but for many of these entities neither the corresponding kinase nor the precise function behind their phosphorylation is clear. The calcium sensing receptor (CAS; At5g23060) is a thylakoid-localized phosphoprotein of unknown function. It has a proposed role in mediating stromal and cytoplasmic Ca^2+^ signals in connection with stomatal movement and immunity-related gene expression in Arabidopsis (Nomura et al., 2008; Weinl et al., 2008; Nomura et al., 2012). Phosphorylation of CAS has been described both as a Ca^2+^-dependent (Stael et al., 2012) as well as a high light-induced, STN8-dependent event (Vainonen et al., 2008), but the functional consequences of this reversible modification remain elusive. *In silico* topology predictions suggest that CAS possesses a single transmembrane domain that splits the protein in two halves of about equal size. Apart from the chloroplast targeting sequence, the N-terminal domain does not possess any identifiable functional characteristics but was described to be able to bind Ca^2+^ *in vitro* with low affinity and high capacity (Han et al., 2003; Wang et al., 2016). The C-terminal domain contains a so-called “non-catalytic rhodanese homology domain” as well as all of the described phosphorylation sites.

In the present work, we investigated the phosphorylation profile of CAS by dissecting the potential contribution of different phosphorylation sites and the involvement of the major thylakoid-localized kinases STN7 and STN8 in its reversible modification. Bioinformatics and phosphoproteomics analyses identified several evolutionary conserved phosphorylation sites and *in vitro* kinase assays provided further evidence of multiple phosphorylation events affecting CAS. Characterization of *cas* mutant plants by analysis of chlorophyll fluorescence emission at 77K revealed similarities to the photosynthetic mutants *tap38/pph1* and *pgr5* (Mekala et al., 2015). Altogether, our results suggest a role of CAS in the STN7/STN8/TAP38-dependent photoacclimation network.

## Materials and Methods

### Plant Material and Growth Conditions

Unless otherwise stated *Arabidopsis thaliana* wild type (ecotype Col-0) and previously described mutant lines *cas-1* (SALK_070416; Nomura et al., 2008; Weinl et al., 2008), *stn7* (SALK_073254; Bellafiore et al., 2005), *stn8* (SALK_060869; Vainonen et al., 2005), and *stn7/8* double mutant (Fristedt et al., 2009) were grown on soil in a growth chamber (equipped with Philips TLD 18W of alternating 830/840 light color temperature) under a 16 h/8 h day/night regime with 100 μmol photons m^–2^ s^–1^.

### Isolation of Protein Extracts From Chloroplast Subfractions

Intact chloroplasts were purified from 4 weeks old Arabidopsis plants as previously described (Seigneurin-Berny et al., 2008) starting from leaf material that was harvested at the end of the dark period or after 4 h in growth light (GL). Intact chloroplast pellets were frozen in liquid nitrogen and stored at −80°C if not used immediately. Thylakoid membrane and stromal protein fractions were obtained as previously described (Rocha et al., 2014) by disrupting intact chloroplasts in lysis buffer (20 mM Tricine/HCl, pH 7.6, 10% (v/v) glycerol and 1 mM DTT) supplemented with protease inhibitors (complete^TM^, EDTA-free; Roche, Mannheim, Germany) and, depending on the type of experiment, with phosphatase inhibitors (Phospho-Stop; Roche, Mannheim, Germany). After incubation on ice for 15 min, membranes and soluble components were separated by centrifugation at 20,000 *g* for 10 min and the thylakoid fraction was washed several times with lysis buffer. All procedures were carried out at 4°C.

Protein concentration of protein extracts was determined by using the Coomassie Bradford protein assay kit (Life Technologies, Darmstadt, Germany) according to the manufacturer’s instructions. Chlorophyll concentration was determined as previously described (Arnon, 1949). Purity of the fractions was confirmed by SDS-PAGE and Western Blot analysis using antibodies against transketolase (α-TKL), fructose 1,6-bisphosphatase (α-FBP), 33 kDa subunit of the oxygen evolving system (α-OE33) and ATP-Synthase (α-ATPase).

### Cloning and Purification of Recombinant CAS Constructs

The CAS-C construct, ranging from AA 216 to 387 of the CAS protein (At5g23060), was the same as used in Stael et al. (2012). All non-phosphorylatable CAS-C variants (CAS-C_T376V_, CAS-C_*S378A*_, CAS-C_T380V_) were obtained via QuickChange site directed mutagenesis (Zheng et al., 2004) on the original CAS-C construct (for a list of primers see Supplementary Table S1). PCR reaction products were treated with *DpnI* enzyme to digest parental vector DNA and transformed in *E. coli* DH5α cells for plasmid amplification.

The CAS-N fragment was cloned in-frame with an N-terminal intein tag into the pTWIN1 vector (New England Biolabs). To this end, the sequence corresponding to amino acidic positions 34–147 of CAS was obtained by PCR from *Arabidopsis thaliana* cDNA using primers containing the restriction sites for *Nco*I and *Pst*I (Supplementary Table S1).

Recombinant CAS-C and CAS-N fragments, including the non-phosphorylatable CAS-C variants, were expressed in *E. coli* strain ER2566 cells and purified under native conditions using the IMPACT^TM^-TWIN system (New England Biolabs, Frankfurt, Germany) following the manufacturer’s instructions. Protein concentration of purified recombinant proteins was determined by using the Coomassie Bradford protein assay kit.

For expression of CAS-YFP and CAS_34–387_-YFP in tobacco mesophyll cells, the full-length CAS coding sequence and a truncated variant lacking the first 33 amino acids corresponding to the predicted transit peptide were cloned N-terminally to the YFP sequence into the plant expression vector pBIN19 (Datla et al., 1992). For self-assembly green fluorescent protein (saGFP) analysis (Cabantous et al., 2005), the full-length coding sequences of CAS and the small subunit of RUBISCO were cloned into the pBIN19-saGFP_1–10_ and pBIN19-saGFP_11_ vectors as described in Ruge et al. (2016).

A list of all construct used in this study is provided in Supplementary Table S2.

### *In vitro* Phosphorylation Assays

*In vitro* phosphorylation of recombinant CAS-C fragments were conducted as previously described (Stael et al., 2012) using ∼200 ng of CAS-C substrates and catalytic amounts (equivalent to ∼2 μg of chlorophyll) of thylakoid membranes. All assays were carried out for 10 min (unless stated otherwise) at room temperature (22°C) in a total volume of 25 μl in kinase buffer containing 20 mM Tricine/HCl, pH 7.6, 10 mM MgCl_2_, 10% (v/v) glycerol, 1 mM DTT, 5 μM ATP and 70–180 kBq of [γ^32^-P]ATP (PerkinElmer, Waltham, MA, United States). Depending on the experiment, assays were conducted at ambient light or in dark and supplemented with 1 μM CaCl_2_ or 2 mM EGTA. Reactions were stopped by the addition of SDS solubilization buffer (Laemmli, 1970) and boiling for 2 min at 96°C. Reaction products were separated via SDS-PAGE, followed by Coomassie staining and drying of the gel. Phosphorylation signals were revealed by exposing dried gels to X-ray films (Carestream^®^ Kodak^®^ X-Omat LS film, Rochester, NY, United States) followed by film processing and development (AGFA G153 developer, Fix AG fixer, Mortsel, Belgium).

### Immunoblot Analysis

Custom polyclonal primary antibodies were generated in rabbit against purified CAS-C and CAS-N fragments (Davids Biotechnologies, Regensburg, Germany). The α-OE33 antiserum was a kind gift from Prof. J. Soll (LMU Munich). The α-pThr antibody was purchased from Cell Signaling (Danvers, MA, United States) and used as previously described (Mekala et al., 2015). Proteins were separated on 10% SDS-PAGE gels and visualized by western blotting using an anti-rabbit secondary antibody coupled to horseradish peroxidase (Sigma-Aldrich, St. Louis, MO, United States), an enhanced chemiluminescence kit (SERVALight EOS, SERVA, Heidelberg, Germany) and the Chemidoc Imaging System (BioRad).

For immune detection-based topology analysis of CAS, a protease treatment of isolated thylakoids was performed prior to SDS-PAGE separation. Isolated thylakoid membranes were resuspended in 0.1 M sucrose, 10 mM HEPES-NaOH, pH 8.0 at 0.5 mg chlorophyll/ml. Depending on the sample, the detergent Triton X-100 was included to a concentration of 1% before the addition of the protease. Untreated and detergent-treated thylakoid samples were incubated with thermolysin (Sigma-Aldrich, St. Louis, MO, United States) in a concentration of 100 μg/ml with 1 mM CaCl_2_ for 20 min on ice. The digestion reaction was stopped by addition of 20 mM EDTA and 4× SDS-sample buffer followed by boiling for 2 min at 96°C. The samples were analyzed by SDS-PAGE and western blot using antibodies against CAS-C, CAS-N, and OE33.

### Confocal Laser Scanning Fluorescence Microscopy

*Agrobacterium*-mediated transient transformation of tobacco leaf cells was performed as previously described (Voinnet et al., 2003). Infiltrated leaves with transiently expressed and co-expressed proteins of interest were harvested after 48 h and used for protoplast isolation (Koop et al., 1996). The subcellular localization of protein was analyzed with a Leica TCS SP5 confocal laser scanning microscope (Leica Microsystems, Germany) using excitation with the 488 nm line of an argon laser for GFP, YFP and chlorophyll. Emission spectra were recorded from 496 to 523 nm (GFP, YFP) and 680 to 713 nm (chlorophyll). Images were taken with a HCX PL APO CS 100.0 × 1.46 OIL objective and image processing was performed using the Leica Application Suite for Advanced Fluorescence (LAS AF) software.

### Analysis of Chlorophyll Fluorescence Emission at 77K

Chlorophyll fluorescence emission spectra were recorded at 77K from frozen thylakoids. Thylakoid membranes were extracted as previously described (Tikkanen et al., 2006) from leaves of 3 weeks old Arabidopsis plants after the following light treatments: end of night (dark, D), 2 h of GL (100 μmol photons m^–2^ s^–1^, alternating Osram Luminux 35W/840 and Philips TLD 18W/860; GL) and subsequent 2 h of high light (1000 μmol photons m^–2^ s^–1^, LED of 3000 K; HL). Briefly, leaves were flash frozen in liquid nitrogen and grinded into powder in a homogenization buffer containing 50 mM Hepes/KOH, pH 7.5, 330 mM sorbitol, 2 mM EDTA, 1 mM MgCl_2_, 5 mM ascorbate, 0.05% (w/v) BSA and 10 mM of the phosphatase inhibitor NaF. All steps were performed at 4°C and under dim green light. The material was subsequently filtered through Miracloth and pelleted by centrifugation at 2500 *g* for 4 min at 4°C, followed by a second wash step in homogenization buffer. For measurements, thylakoids were diluted to a chlorophyll concentration of 1 μg ml^–1^ concentration.

Chlorophyll fluorescence emission spectra were measured from frozen thylakoids by exciting the samples at 480 nm and recording the emission spectrum between 650 and 800 nm using an Ocean Optics QE Pro Spectrometer. Three biological and technical replicates for each genotype and treatment were measured. Ratio values of chlorophyll fluorescence emission peaks of PSI over PSII were calculated by manual normalization of the traces to the PSII peak value at 685 nm.

### LC-MS/MS Analysis of Phosphopeptides From Thylakoid Membranes

Intact chloroplasts were isolated as described above from wild type and *stn8* mutant plants after 4 h of GL exposure. Preparation of surface-exposed peptides from thylakoid membranes via trypsin digestion was performed as previously described (Vener and Strålfors, 2005; Rokka et al., 2011). Samples were flash frozen in liquid nitrogen, thawed, and the digestion products were centrifuged for 20 min at 14,000 *g*. Enrichment of released phosphopeptides was performed using 5 mg of TiO_2_ (Glygen Corp.) as described previously (Bodenmiller et al., 2007; Chen et al., 2010). Subsequently, peptides were lyophilized to dryness in a vacuum concentrator.

Phosphopeptide analysis of the lyophilized samples was performed as described in Roustan et al. (2017). MaxQuant 1.5^1^ and the Andromeda search algorithm were used against the TAIR-10 database to perform peptide identification, phosphorylation site mapping and phosphopeptide quantification (Cox and Mann, 2008; Cox et al., 2011). The following stringency criteria were applied in the analysis: three missed cleavages were allowed and methionine oxidation and protein N-terminal acetylation were endorsed as dynamic modifications. Additionally, phosphorylation of serine, threonine and tyrosine residues was permitted to occur as dynamic modifications. Mass tolerance was set to 5 ppm for parental ions and 0.5 Da for the MS/MS fragment. For both peptide and protein levels, false discovery rate was set to 1%. Quantification was done at the peptide level. Perseus 1.5 software was used for further filtering and data processing (Tyanova et al., 2016). Phosphopeptides were accounted for quantification if they could have been quantified in at least 70% of the biological samples. Additionally, only phosphopeptides that passed the class I criteria (phosphosite probability > 75% and score difference >5) were included in the final dataset (Olsen et al., 2006).

### Bioinformatics Analysis

Orthologs of CAS in various organisms were identified by blastp or tblastn searches at NCBI. Alignments were performed using Clustal X 2.0 (Thompson et al., 1997). For dicots, monocots, gymnosperms and green algae consensus sequences were defined based on the full alignments shown in Supplementary Data S1–S4. Some hand-alignment was performed to give what we consider the best fit.

### Accession Numbers

Accession numbers for all sequence data can be found in Supplementary Table S3.

## Results

### Phylogenetic Conservation of CAS Phosphorylation Sites

The state transition kinases STN7 and STN8 are the best-described protein kinases in the thylakoid membrane (Bellafiore et al., 2005; Bonardi et al., 2005; Vainonen et al., 2005; Pesaresi et al., 2011) and the CAS protein was initially described as a potential target of STN8 (Vainonen et al., 2008). At the same time, *in vitro* phosphorylation studies have shown Ca^2+^-dependent phosphorylation of CAS (Stael et al., 2012), indicating that this protein might be a shared target of multiple kinases.

**FIGURE 1:**
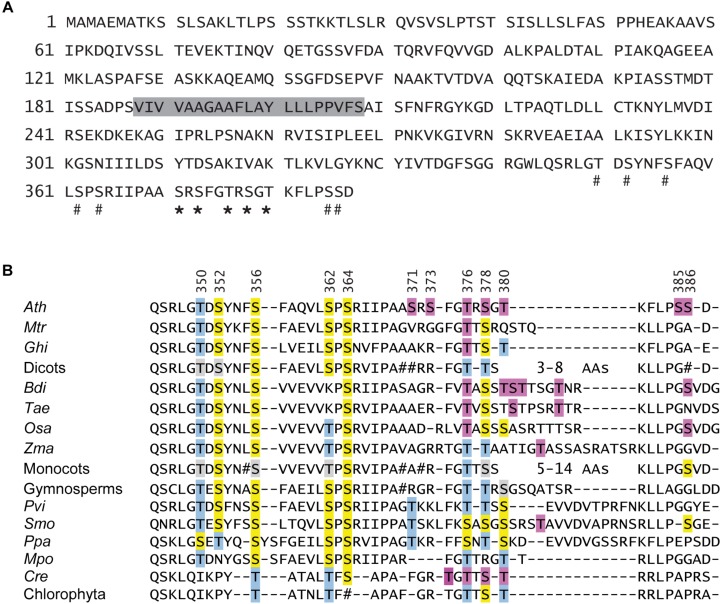
CAS domain structure and phylogenetic conservation of phosphorylation sites. **(A)** Amino acid sequence of the CAS (At5g23060) protein of *A. thaliana*. The predicted transit sequence and the transmembrane domain are marked by a solid line and a gray box, respectively. Thr and Ser residues that were unambiguously identified by phospho-proteomic studies are marked by an asterisk, while sites that are part of a phosphopeptide (tentative sites) are marked by a hash. **(B)** Sequence alignment of the CAS C-terminal domain from organisms throughout the *Viridiplantae* clade. For dicots, monocots, gymnosperms and green algae, a consensus sequence was created based on alignments shown in Supplementary Data S1–S4. Those organisms, for which phosphoproteomics data on CAS are available, are listed separately. Residues for which phosphorylation was shown unambiguously are indicated by a purple box. For Arabidopsis, five more Ser and Thr residues are present in phosphopeptides for which the exact phosphoresidue was not determined. These and all Ser and Thr residues that can be aligned to potential pSer or pThr from Arabidopsis CAS are marked in yellow or blue, respectively. Positions where a majority but not all sequences from a consensus have a Ser or Thr are marked in light gray. Positions where no clear conservation (<70%) was observed in a consensus are marked by #. In many sequences from the mono- and dicots, a stretch of variable length is found toward the C-proximal end. In all cases this part contains at least one Ser or Thr residue. *Ath, A. thaliana; Mtr, M. truncatula; Ghi, G. hirsutum; Bdi, B. distachyon; Tae, T. aestivum; Osa, O. sativa; Zma, Z. mays; Pvi, P. vittata; Smo, S. moellendorffii; Ppa, P. patens; Mpo, M. polymorpha; Cre, C. reinhardtii.*

Vainonen and co-workers suggested Thr-380 as the site of STN8 phosphorylation (Figure 1A). However, further experimental studies (mostly in the form of large-scale phosphoproteomics analyses; Table 1) have indicated multiple phosphorylated Thr and Ser residues (Heazlewood et al., 2008; Durek et al., 2010), the majority of which are located close to the C-proximal end of CAS. For seven positions, the phosphoresidue was identified unequivocally (Figure 1A, asterisks; Table 1), while in all other cases the precise position of the modification within an identified phosphopeptide was not assigned (Figure 1A, hash).

**TABLE 1:** Phosphopeptides identified for CAS proteins from various organisms.

**Species**	**Phosphosite**	**Corresponding site in *A. thaliana***	**Phosphosite(s) or phosphopeptide**	**References**
*A. thaliana*	Thr-350; Ser-352; Ser-362; Ser-364		LGTDSYNFSFAQVLSPSR	a, b
	**Ser-371**		IIPAA**pS**R**pS**FGTR IIPAA**pS**R**pS**FG**pT**R	g, h
	**Ser-373**		IIPAA**pS**R**pS**FGTR IIPAA**pS**R**pS**FG**pT**R IIPAASR**pS**FG**pT**R **pS**FGTRSGTKFLPSSD **pS**FGTR**pS**GTKFLPSSD **pS**FGTRSG**pT**KFLPSSD	a–c, f–h
	**Thr-376**		IIPAASR**pS**FG**pT**R IIPAA**pS**R**pS**FG**pT**R SFG**pT**RSGTKFLPSSD SFG**pT**RSG**pT**KLPSSD	b, f, h this work
	**Ser-378**		SFGTR**pS**G**pT**K **pS**GTKFLPSSD **pS**FGTR**pS**GTKFLPSSD	a–f, h this work
	**Thr-380**		SFGTRSG**pT**KFLPSSD **pS**FGTRSG**pT**KFLPSSD SFG**pT**RSG**pT**KLPSSD SG**pT**KFLPSSD SFGTR**pS**G**pT**K	c, f–i this work
	Ser-385; Ser-386		SGTKFLPSSD	b
*M. truncatula*	**Thr-378**	**Thr-376**	GGFG**pT**TSR	J
*G. hirsutum*	**Thr-390**	**Thr-376**	FG**pT**TSTK	k
*B. distachyon*	**Thr-369**	**Thr-376**	FV**pT**ASS**pT**pSTTSGTNR FV**pT**ASSTS**pT**TSG**pT**NR	m, n
	**Thr-373**	**Thr-380**	FV**pT**ASS**pTpS**TTSGTNR	m
	**Ser-374**	-/-	FV**pT**ASS**pTpS**TTSGTNR	m
	**Thr-375**	-/-	FV**pT**ASSTS**pT**TSG**pT**NR	n
	**Thr-379**	-/-	FV**pT**ASSTS**pT**TSG**pT**NR	n
	**Ser-387**	**Ser-386**	KLLPG**pS**VDG	m, n
*T. aestivum*	**Thr-368**	**Thr-376**	FV**pT**VSSTSTPSR**pT**SR	l
	**Ser-373**	-/-	FV**pT**VSST**pS**TPSRTTR	l
	**Thr-378**	-/-	FV**pT**VSSTSTPSR**pT**SR	l
*O. sativa*	**Ser-384**	**Ser-386**	KLLPG**pS**VDG	m
	**Thr-366**	**Thr-376**	LV**pT**ASSSASR	n
*Z. mays*	**Thr-377**	-/-	IG**pT**ASSASR	p, q
*C. reinhardtii*	**Thr-365**	-/-	GR**pT**G**pT**TSTR	r
	**Thr-367**	**Thr-376**	GR**pT**G**pT**TSTR TG**pT**TS**pT**RRLPAPR	r
	**Ser-369**	**Ser-378**	TG**pT**TS**pT**RRLPAPR	r
	**Thr-370**	**Thr-380**	TGTT**pSpT**RRLPAPR	r

*Unambiguously identified phosphoresidues are indicated in bold. Accession numbers can be found in Supplementary Table S3. (a) Reiland et al. (2009), (b) Reiland et al. (2011), (c) Nguyen et al. (2012), (d) Hoehenwarter et al. (2013), (e) Wang et al. (2013), (f) Yang et al. (2013), (g) Lin et al. (2015), (h) Roitinger et al. (2015), (i) Bhaskara et al. (2017); (j) Rose et al. (2012); (k) Fan et al. (2014); (l) Lv et al. (2014a), (m) Lv et al. (2014b), (n) Lv et al. (2014c); (o) Whiteman et al. (2008); (p) Bonhomme et al. (2012), (q) Fristedt et al. (2012), (r) Wang et al. (2014).*

We performed a comparative analysis of CAS orthologs to investigate the phylogenetic conservation of the phosphosites described for Arabidopsis. Proteins with significant sequence similarity to CAS can be found in all organisms of the *Viridiplantae* clade for which complete genomes or substantial genomic data are available (Supplementary Table S4). For groups such as dicots, monocots, gymnosperms and green algae, where multiple sequences are available, consensus sequences were obtained using Clustal X on which the data in Figure 1B are based. For ferns, only a single complete sequence was available from *Pteris vittata* (Pvi) and in case of the bryophytes, the sequences from *Physcomitrella patens* (Ppa) and *Marchantia polymorpha* (Mpo) deviated in their C-terminus so much that a consensus could not be made. *Medicago truncatula* (Mtr) and *Gossypium hirsutum* (Ghi; dicots), *Oryza sativa* (Osa), *Zea mays* (Zma), *Triticum aestivum* (Tae) and *Brachypodium distachyon* (Bdi; monocots), *Selaginella moellendorffii* (Smo; early diverging lineage of vascular plant) as well as *Chlamydomonas reinhardtii* (Cre; green algae) are listed individually because phospo-peptide data on CAS from these organisms are available (Table 1). Of note, no CAS orthologs were identified in cyanobacteria, red algae or glaucophyta.

The evolutionary conservation of all 12 Ser and Thr residues found within the C-proximal end of the Arabidopsis CAS protein is represented in Figure 1B. Residues for which phosphorylation was shown unambiguously independent of the organism are indicated by a purple box. For Arabidopsis, five more Ser and Thr residues are part of phosphopeptides for which the exact phosphoresidue position was not determined. These and all Ser and Thr residues that can be aligned to proven and potential pSer or pThr from Arabidopsis CAS (see also Table 1) are marked in yellow or blue, respectively, in Figure 1B. Up to Ser-371 in the Arabidopsis sequence, all CAS sequences show a quite high degree of similarity. Unfortunately, from there onwards the sequence of CAS proteins becomes much less conserved and only the very proximal end of about 7 amino acids can be reasonably well aligned. In between, the sequences vary between one threonine in *P. patens* up to 14 amino acids in certain monocots. The best fit that we could create for this region suggests a strong evolutionary conservation of Thr-376 and this site can be aligned with good confidence to phosphoresidues identified in *C. reinhardtii*, *O. sativa*, *B. distachyon*, *G. hirsutum*, and *M. truncatula* (Figure 1B and Table 1). A somewhat similar level of conservation can be observed for Thr-378, which is represented by a Ser or Thr residue in the vast majority of sequences. Instead, Thr-380 shows a lower degree of conservation but seems to be represented by a Ser or Thr residues in many sequences. Also, in the highly variable and therefore difficult to align C-terminal part of most monocot and dicot sequences, at least two Ser or Thr residues can be found that could functionally substitute Thr-380 of Arabidopsis. Indeed, phosphorylated Thr or Ser residues within this variable region were detected in organisms as divergent as *B. distachyon*, *Z. mays, S. moellendoerffii*, and *C. reinhardtii* (Table 1). Together, these data indicate a strong evolutionary conservation of some CAS phosphorylation sites that goes back to the origin of the plant cell. Other potential phosphorylation sites show different degrees of phylogenetic conservation. Notably, Ser-371 and Ser-373, which were unambiguously shown as phosphorylated in Arabidopsis (Table 1), are not conserved at all, indicating that there might exist a certain level of organism-specific phosphorylation pattern(s) of CAS.

### CAS Is a Target of Both State Transition Kinases

When a phosphoproteomics analysis was performed on growth light (GL; 100 μmol photons m^–2^ s^–1^) acclimated Arabidopsis wild type and *stn8* mutant plants, we found three of the previously described phosphorylation sites, namely Thr-376, Ser-378, and Thr-380 (Supplementary Table S4 and Supplementary Data S5). Phosphorylated versions of all three residues were detectable in wild type and the *stn8* mutant, albeit at lower abundance, indicating that they are target sites of STN8 but can still be phosphorylated in its absence. This is in agreement with Thr-380 having previously been found phosphorylated in the *stn7* and *stn8* single mutants but not in the *stn7/8* double mutant (Ingelsson and Vener, 2012). In our analysis, proteins that are not targets of STN8 (Reiland et al., 2011) showed no difference in phosphorylation (Supplementary Table S4), supporting that the reduced levels of CAS phosphorylation are indeed due to the lack of STN8.

**FIGURE 2:**
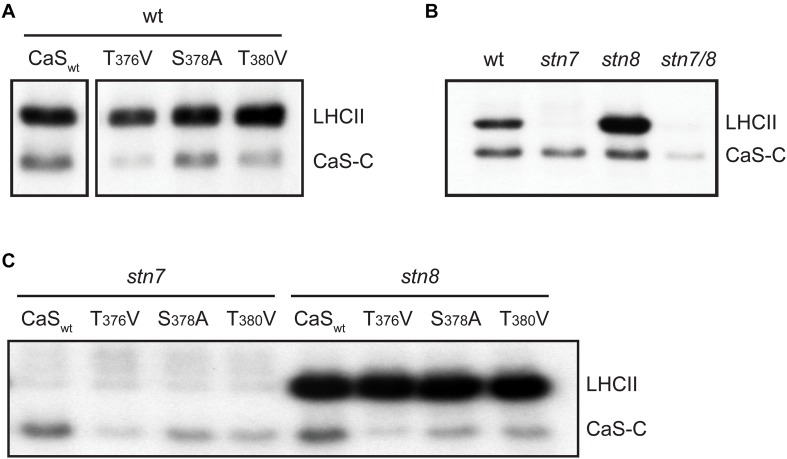
*In vitro* phosphorylation of a recombinant CAS fragment (CAS-C) by thylakoid extracts from wild type, *stn7*, *stn8*, and *stn7/8* plants. **(A)** CAS-C (CaS_wt_) and variants with altered Ser and Thr residues were phosphorylated using thylakoid extracts from wild type in the presence of Ca^2+^. Phosphorylation of endogenous LHCII is also visible. **(B)** Phosphorylation assays with the CAS-C fragment were performed in the presence of Ca^2+^ using thylakoid extracts from wild type (wt), *stn7*, *stn8*, and *stn7/8* double mutant plants. **(C)** CAS-C (CaS_wt_) and variants with altered Ser and Thr residues were phosphorylated in the presence of Ca^2+^ using thylakoid extracts from *stn7* and *stn8* mutant plants. All panels show representative results of experiments performed several times. Corresponding Coomassie stained gels for all panels are shown in Supplementary Figure S2.

We next performed *in vitro* phosphorylation assays using a 171 amino acid long recombinant fragment of CAS (CAS-C) that contains all phosphoresidues determined by different phosphoproteomics studies (AAs 216-387). We generated three non-phosphorylatable variants of the CAS-C fragment by replacing threonine (at Thr-376 and Thr-380) and serine (at Ser-378) residues with valine and alanine, respectively. *In vitro* phosphorylation assays were conducted using thylakoid membranes from GL-adapted wild type plants in the presence of 1 μM Ca^2+^ (unless stated otherwise) to promote full activation of all potentially involved kinases. In agreement with the phosphoproteomics results, all three variants showed a reduction in phosphorylation compared to the wild type construct (Figure 2A and Supplementary Figure S1). The reduction for CAS-C_*S378A*_ was only minor, while the strongest reduction was observed for CAS-C_T376V_. This observation is in contrast with the notion that Thr-380 is the major phosphorylation site of CAS (Vainonen et al., 2008). However, the phosphopeptide detected in that study only started with Arg-377 and thus did not include Thr-376.

Since the phosphoproteomics analysis indicated that CAS is still phosphorylated in the absence of STN8, the *in vitro* phosphorylation of CAS-C was investigated by comparing thylakoid membranes derived from Arabidopsis wild type, *stn7*, *stn8*, and *stn7/8* mutants (Figure 2B and Supplementary Figure S1). As expected, endogenous LHCII, which is the major target of STN7 (Bonardi et al., 2005), is strongly phosphorylated in the wild type and the *stn8* mutant, while very little phosphorylation is evident in the *stn7* and *stn7/8* mutants. By contrast, phosphorylation of recombinant CAS-C was observed in both single mutants, but was mostly abolished in the *stn7/8* mutant. Similar to endogenous LHCII protein, phosphorylation of CAS-C also appears slightly enhanced in the *stn8* mutant. An increased STN7 activity on LHCII in the absence of STN8 can be observed in similar studies (Bonardi et al., 2005; Tikkanen et al., 2010) and could be due to a compensatory mechanism affecting all targets of STN7, especially if they are shared with STN8. Thus, in line with the phosphoproteomics data, these results show that CAS is not an exclusive target of STN8 but can also be phosphorylated by STN7. When the three phosphosite variants of CAS-C were incubated with thylakoids originating from GL-acclimated *stn7* and *stn8* plants, a similar pattern of phosphorylation reduction as in the wild type was observed for both mutants, suggesting that the two STN kinases do not target different phosphorylation sites of CAS (Figure 2C and Supplementary Figure S1).

### CAS C-Terminal Phosphorylation Sites Are Exposed to the Stroma

Phosphorylation of CAS-C by STN7 and STN8 requires the accessibility of the CAS C-terminus to the active site of these kinases. In the initial paper describing CAS, and in accordance with *in silico* topology predictions, a single transmembrane-spanning domain was suggested that splits the protein into two nearly equal parts (Figure 1A, gray boxing). When the thylakoid localization of CAS and its phosphorylation by STN8 were described (Vainonen et al., 2008), it was concluded that the C-terminal domain has to be exposed to the stroma. However, while stromal exposure of the N-terminus could be confirmed by western blot analysis using an antibody directed against the N-terminus of CAS (Nomura et al., 2008), the localization of the C-terminus was never proven experimentally.

**FIGURE 3:**
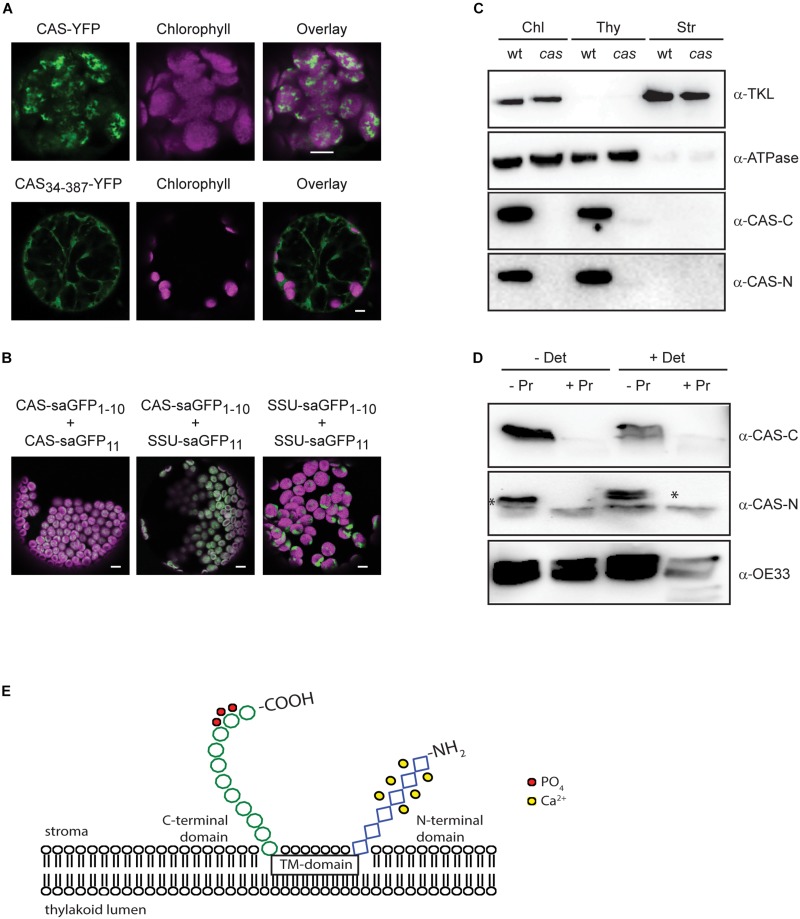
Topology analysis of CAS. **(A)** Expression of CAS-YFP in tobacco protoplasts shows a clear overlap of the YFP and chlorophyll fluorescence signal confirming the correct targeting of CAS-YFP into the chloroplast. Removal of the predicted transit peptide (CAS_34–387_-YFP) resulted in a cytosolic localization (white bars indicate 5 μm). **(B)** Fluorescence analyses of tobacco leaf cell protoplasts co-transformed with the self-assembly GFP pairs CAS-saGFP_1–10_/CAS-saGFP_11_, CAS-saGFP_1–10_/RUBISCO-saGFP_11_, and RUBISCO-saGFP_1–10_/Rubisco-saGFP_11_ confirm that the C-terminus of CAS is exposed to the stromal side of the thylakoid membrane (white bars indicate 5 μm). **(C)** Isolated chloroplasts from wild type (wt) and *cas* mutant plants were separated into thylakoid membranes and stroma and all fractions were probed with antibodies directed against the two CAS domains (α-CAS-C; α-CAS-N), beta-subunit of the chloroplast ATP-synthase (α-ATPase) and transketolase (α-TKL). A corresponding Coomassie-stained gel is shown in Supplementary Figure S3. **(D)** Isolated thylakoid membranes from wild type plants were treated with the protease thermolysin (+/− Pr) in the absence or presence of detergent (+/− Det). All fractions were probed with antibodies against the C- and N-terminal domain of CAS (α-CAS-C, α-CAS-N) as depicted in Figure 1A. The asterisk indicates the specific reaction of the α-CAS-N antibody with CAS. An antibody against the oxygen-evolving system protein 33 (α-OE33) was used as a control to assess the integrity of the thylakoid membrane during the treatment. **(E)** Topology model of CAS showing the exposure of conserved phosphorylation sites (red circles) onto the stromal surface of the thylakoids. Ca^2+^-binding to the stromal exposed N-terminal domain is indicated by yellow circles.

We thus analyzed the topology of CAS with two different experimental approaches. After confirming the correct chloroplast targeting of a CAS-YFP fusion construct in the pBIN19-based vector system (Mehlmer et al., 2012) by transient expression in *N. benthamiana* leaves (Figure 3A), we employed a pBIN19-based self-assembling GFP (saGFP) system, which had been previously used to study the topology of membrane proteins (Cabantous et al., 2005; Machettira et al., 2011). We fused CAS or the small subunit of the Ribulose-1,5-bisphosphate carboxylase/oxygenase (SSU) to either the 11th beta sheet (CAS-saGFP_11_; SSU-saGFP_11_) or the first 10 beta-sheets (CAS-saGFP_1–10_; SSU-saGFP_1–10_) of GFP. Assembly of a functional GFP occurs when both parts are present in the same cellular compartment. Co-infection of CAS-saGFP_1–10_ with CAS-saGFP_11_ and SSU-saGFP_1–10_ with SSU-saGFP_11_ resulted in GFP fluorescence clearly overlapping with the chlorophyll fluorescence, indicating that both proteins are correctly targeted into the chloroplast (Figure 3B). The same result was achieved when CAS-saGFP_1–10_ was co-infected with SSU-saGFP_11._ Since SSU is a soluble stromal protein, these results strongly support an extrusion of the C-terminal domain of CAS into the stroma.

We furthermore generated polyclonal antibodies against the N-terminal (AA 34–147; α-CAS-N) and the C-terminal domain (AA 216–387; α-CAS-C) of CAS. Both antibodies were able to recognize their recombinant antigen and did not show any cross-reactivity with the other domain (Supplementary Figure S3). They both recognize a protein of about 38 kDa in chloroplast extracts and this reaction was absent in the *cas* mutant line (Figure 3C and Supplementary Figure S4). When tested on different protein fractions, i.e., total chloroplasts, thylakoid membranes, and stromal/soluble proteins, CAS was only detected in chloroplast extracts and the thylakoid membrane fraction (Figure 3C), corroborating its predicted localization. We then treated isolated thylakoid membranes with thermolysin to remove those parts of thylakoid membrane proteins that extrude into the stroma. Probing of untreated and treated samples with an antibody against the thylakoid lumen protein OE33 revealed no loss of reactivity after thermolysin digestion, indicating that the thylakoid membrane remained intact throughout the treatment (Figure 3D, α-OE33). Thermolysin-mediated degradation of OE33 could only be achieved when a detergent was added to the thylakoid membranes along with the protease. By contrast, no signal was detected in thermolysin-treated thylakoids when probed with antisera against either α-CAS-C or α-CAS-N (Figure 3D). These results corroborated the orientation of both termini toward the stromal side of the thylakoid membrane thereby giving the C-terminal phosphorylation sites access to the active domains of STN7 and STN8 (Figure 3E).

### CAS Is Required for Acclimation to High Light

**FIGURE 4:**
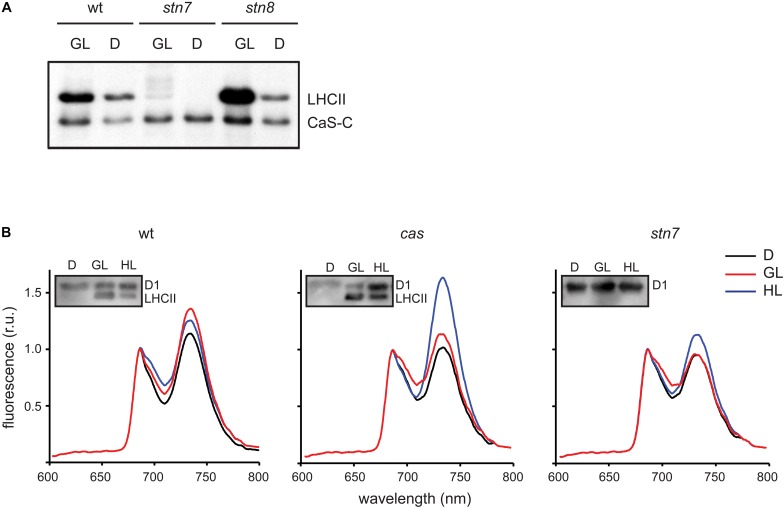
CAS phosphorylation and 77K chlorophyll fluorescence emission spectra of thylakoid membranes harvested under different light conditions. **(A)** *In vitro* phosphorylation assays were performed with the CAS-C fragment using thylakoid extracts from wild type (wt), *stn7, and stn/8* mutant plants harvested in the dark (D) or the growth light (GL) period. **(B)** Chlorophyll fluorescence emission spectra were recorded at 77K using thylakoid membranes isolated from wild type (wt), *cas*, and *stn7* mutant plants harvested in the dark (D), after 2 h of GL (100 μmol photons m^–2^ s^–1^, GL) and 2 h of high light (1000 μmol photons m^–2^ s^–1^, HL). Samples from the same thylakoids were probed with an α-pThr antibody to assess the phosphorylation status of the LHCII complex and the D1 protein (inlays).

The work of Vainonen and coworkers suggested a light-dependent regulation behind the *in vivo* phosphorylation of CAS (Vainonen et al., 2008). In line with their suggestion, a clear difference in the *in vitro* phosphorylation intensity of recombinant CAS-C was evident in this work when GL-adapted thylakoid membranes from wild type plants were compared with dark-harvested ones (Figure 4A and Supplementary Figures S4, S5). A similar difference could be observed in the phosphorylation of the endogenous LHCII, reflecting the light-dependent activation of STN7 (Bellafiore et al., 2005). As expected, no LHCII phosphorylation was observed in the *stn7* mutant, while its phosphorylation was slightly enhanced in the *stn8* mutant. For CAS-C, an impact of the light treatments on its phosphorylation was evident in case of the *stn8* mutant but not in *stn7*, indicating that the light-dependent difference observed in the wild type is mediated by the activity of STN7.

The role of STN7 is strongly associated with the regulation of energy distribution between PSI and PSII. In this work, when chlorophyll fluorescence emission spectra at 77K were measured from isolated thylakoid membranes of wild type plants, they showed a typical increase in the PSI peak at 733 nm upon shift from darkness (state 1) to GL (state 2), as visible from both a representative graph (Figure 4B) and the averaged peak values of three biological replicates (Table 2). As described previously (Tikkanen et al., 2010; Mekala et al., 2015), a slight reduction of the PSI peak occurred after a subsequent 2 h of high light (1000 μmol photons m^–2^ s^–1^) treatment (Figure 4B and Table 2; wt), reflecting the disassociation of the mobile LHCII antenna from PSI. The *cas* mutant also showed an increase in the PSI peak upon transition into GL, indicating no major impairment in performing light-induced state 1- state 2 transition. The *stn7* mutant behaved according to what has been described before, showing an inability to perform phosphorylation-mediated state transition, as visible by an equal PSI peak height in dark and GL conditions (Figure 4B and Table 2; *stn7*). However, *cas* plants did not resemble the *stn7* mutant but showed a slight increase in the PSI peak height between dark and GL and, remarkably, a sustained increase of the PSI peak upon subsequent transfer to high light (Figure 4B and Table 2; *cas*), suggesting a persistent strong excitation of PSI under this condition. A similar phenotype had been observed for the *tap38/pph1* and the *pgr5* mutants (Mekala et al., 2015), the former not being able to properly dephosphorylate LHCII under high irradiance, while the latter is impaired in the cyclic electron transport around PSI. When the *in vivo* phosphorylation status of thylakoid proteins was tested following the same light treatments using a pThr-specific antibody, the wild type showed a characteristic increase in the phosphorylation of PSII core protein D1 and decreased LHCII phosphorylation under high light, while the *cas* mutant showed a strong concomitant phosphorylation of both LHCII and PSII core (Figure 4B, inlays; Supplementary Figure S5).

**TABLE 2:** Ratio values of chlorophyll fluorescence emission peaks of PSI (at 733 nm) over PSII measured at 77K with thylakoids from wild type (wt), cas, and stn7 mutant lines.

**Genotype**	**Treatment**	**PSI/PSII peak ratio**
Wt	D	1.09±0.03
	GL	1.29±0.05
	HL	1.2±0.05
*cas*	D	1±0.16
	GL	1.1±0.08
	HL	1.58±0.09
*stn7*	D	1±0.1
	GL	1±0.02
	HL	1.08±0.05

*Thylakoids were isolated during the dark period (D) after 2 h of growth light (GL) or subsequent 2 h of high light (HL). Data are means of three biological replicates ± SD.*

### Ca^2+^-Dependent Phosphorylation of CAS Does Not Involve the STN Kinases

The data shown above suggest a connection between CAS phosphorylation by the thylakoid kinases STN7 and STN8 and photoacclimation, with Thr-376, Thr-380, and Ser-378 as their main and shared targets. However, CAS was also suggested as the target of Ca^2+^-dependent phosphorylation (Stael et al., 2012) via experiments in which whole chloroplast and stroma-enriched fractions were used instead of thylakoid membranes.

**FIGURE 5:**
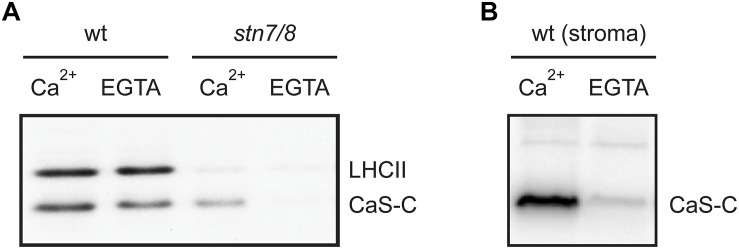
Calcium-dependent phosphorylation of CAS by a stromal kinase. *In vitro* phosphorylation assays with the CAS-C fragment were performed using thylakoid extracts from wild type (wt) and *stn7/8* mutant plants **(A)** or stromal extracts from wild type **(B)** in the presence of either Ca^2+^ or EGTA. All panels show representative results of experiments performed several times. Corresponding Coomassie stained gels for all panels are shown in Supplementary Figure S2.

We thus analyzed the ability of both stroma and isolated thylakoid membranes to phosphorylate CAS-C (Figure 5 and Supplementary Figures S1, S2) either in the presence or absence of Ca^2+^ (1 μM CaCl_2_ or 2 mM EGTA, respectively). In case of wild type thylakoids, only minor differences in the phosphorylation of CAS-C could be observed under either condition (Figure 5A, wt). The residual phosphorylation that could be observed with thylakoids devoid of both STN kinases was completely abolished in the absence of Ca^2+^ (Figure 5A, *stn7/8*). This observation supports the notion that CAS is the target of a Ca^2+^-regulated phosphorylation event, which does not involve STN7 or STN8. When stromal extracts from wild type plants were used in the phosphorylation assay, the Ca^2+^ requirement became much more evident with very little phosphorylation seen in the presence of EGTA (Figure 5B), indicating the presence of a Ca^2+^-dependent kinase in the stroma that targets the stromal exposed C-terminal domain of CAS. The residual Ca^2+^-dependent phosphorylation of CAS in the thylakoid fraction could be due to some minor amounts of this stromal kinase remaining even after washing of the thylakoids. This is supported by the faint band visible for TKL and Fructose 1,6-bisphosphatase in the thylakoid fraction (Figure 3C and Supplementary Figure S6). While this needs to be analyzed in much more detail, there is a clear indication that CAS is a target of kinases other than STN7 and STN8, which is also in accordance with the identification of additional phosphorylation sites that are not targeted by these two kinases.

## Discussion

Understanding the role of chloroplast protein phosphorylation has been hampered by the fact that only few chloroplast kinases have been identified and that plants devoid of the two so far best described kinases, STN7 and STN8, only show surprisingly mild phenotypes. Moreover, the presence of multiple, evolutionary conserved phosphoresidues is a recurring feature of thylakoid phosphoproteins (Grieco et al., 2016), indicating the existence of complex phosphorylation networks involving differential regulation by multiple kinases in relation to specific environmental cues.

Vainonen et al. (2008) pinpointed Thr-380 of CAS as the target of high light-dependent phosphorylation by the STN8 kinase. In the present work, we could confirm the phosphorylation of CAS by STN8 but could also demonstrate the involvement of STN7 and at least one other, so far unknown Ca^2+^-dependent kinase. The orientation of the CAS C-terminus, which contains all known phosphorylation sites, is consistent with the predicted localization of the catalytic domains of STN7 and STN8 that are also exposed to the stroma (Wunder et al., 2013). Any additional kinase acting on CAS would also need to either have its catalytic domain exposed to the stromal surface of the thylakoids or be a stromal protein. Indeed, Ca^2+^-dependent phosphorylation of CAS is much more prevalent when stroma (Figure 2E) or total chloroplast extracts (Stael et al., 2012) are used in the phosphorylation assay instead of isolated thylakoid membranes (Figure 2D), suggesting that the Ca^2+^-dependent activity observed with the thylakoid membrane fractions is due to the residual activity of a stromal kinase. Phosphoproteomic analysis of *stn8* mutant plants revealed a substantial decrease in the relative phosphorylation levels not only of Thr-380 but also Thr-376 and Ser-378 (Supplementary Table S4). *In vitro*, phosphorylation of CAS by either STN7 or STN8 is strongly reduced in the absence of Thr-376 or Thr-380 and to a lesser extent also of Ser-378 (Figures 2A,C). The effect is most pronounced in the absence of Thr-376, which is also the most conserved of those residues for which phosphorylation has been shown unequivocally in Arabidopsis (Figure 1B). Amino acid sequence alignments suggest that the corresponding position was also identified as a phosphoresidue in the green alga *C. reinhardtii*, the monocot *Z. mays* and several dicots (Figure 1B and Table 1). The phosphopeptide identified by Vainonen et al. (2008) study started with Arg-377, which could explain why they did not identify pThr-376. Of note, residue Thr-376 of Arabidopsis, as well as many of the corresponding Thr sites in CAS orthologs included in our phylogenetic analysis (Figure 1B and Supplementary Data S2–S5), are preceded at position -1 by a Gly residue. This configuration matches a minimal phosphorylation consensus motif identified by Schönberg et al. (2017) for newly described targets of STN7. Thr-380 and Ser-378 are also highly conserved and the phosphorylation of a corresponding residue was shown for the green alga *C. reinhardtii*, the lycophyte *S. moellendorffii*, the monocot *Z. mays* and two dicots in case of Thr-380 and in *C. reinhardtii* for Ser-378 (Figure 1B and Table 1). These data indicate that phosphorylation of these three residues is evolutionarily conserved from green algae onwards. For all other potential phosphoresidues the data are much less robust and the corresponding residues display very little conservation.

All proposed phosphoresidues, including Thr-376, Ser-378, and Thr-380, fall within a very confined region found at the very C-proximal end of CAS (Figure 1A). It is thus conceivable that this short amino acidic sequence constitutes a hub for the integration of multiple signals in the form of differential phosphorylation events. Phosphorylation could either occur simultaneously on several residues or on individual sites following the activation of distinct protein kinases in response to cues of very diverse origin, such as light signals in the case of the STN kinases, or local increases in Ca^2+^ concentration with respect to the still uncharacterized Ca^2+^-dependent kinase. The reaching of a certain phosphorylation threshold might be required to fully promote the functional effects of the modification. Alternatively, it could be envisioned that combinatorial, stimulus-dependent phosphorylation events on CAS lead to various, phosphocode-dependent outcomes impacting both developmental as well as stress-related functions of CAS.

Ca^2+^ is best described for its role as second messenger that transduces abiotic and biotic stress signals into a cellular response. However, chloroplasts also display a well-described light-off Ca^2+^ transient and it has been shown that the *cas* mutant is impaired in chloroplast Ca^2+^ responses to elicitors, heat and the light–dark transition (Nomura et al., 2012; Lenzoni and Knight, 2018). Consequently, a potential role of CAS in regulating photosynthetic function could be envisioned by either influencing chloroplast Ca^2+^ dynamics and/or by being differentially phosphorylated in a Ca^2+^-dependent fashion.

In only few of the phosphoproteomic studies listed in Table 1, the light conditions upon harvesting of the samples are described. However, if not otherwise stated, harvesting of the samples during the light period is likely. Thus, in agreement with the results from Vainonen et al. (2008) and because it has been detected as a phosphoprotein in most if not all phosphoproteomics studies of plant proteins, it is likely that CAS is constitutively phosphorylated during daytime. Yet, its overall phosphorylation status and specific phosphorylation profile might subtly change in response to specific environmental cues that promote the activation of different kinases or the opposing phosphatases. It could be envisioned that at early and late day times, characterized by a low light environment, CAS is primarily targeted by the STN7 kinase, leading to a low level of steady-state phosphorylation, which might be required to achieve an optimal excitation balance between PSI and PSII. At midday, when peak irradiance is reached, the STN8 kinase could become more relevant and increase the overall CAS phosphorylation (Vainonen et al., 2008). This situation would place CAS as another shared target of the STN kinases (Bonardi et al., 2005) and their cooperative, differential, or even spatially segregated activity on CAS would dictate the functional outcome(s) of its modifications. The similarity of the *cas* mutant to *tap38* with regard to the 77K chlorophyll fluorescence emission spectra profiles suggests that CAS might be required to promote acclimation responses to excess light, which include the reversal of LHCII phosphorylation (Rintamaki et al., 1997). A phosphorylated form of CAS under the control of STN8 might in fact physically interact or indirectly influence the activity of the TAP38 phosphatase. Alternatively, phosphorylation of CAS by STN8 under high irradiance might negatively regulate the activity of the STN7 kinase, leading to attenuated LHCII phosphorylation and to the physical dissociation of the mobile antenna fraction from PSI reaction centers. In the *cas* mutant such inhibitory effect would be absent and a fully active STN7 would result in PSI being “locked” in state 2 under high light. This situation could explain the impaired PSI de-excitation kinetics observed in the *cas* mutant following excess light treatment. Considering the additional resemblance of the 77K chlorophyll fluorescence emission spectra of *cas* with that of *pgr5*, an involvement of Arabidopsis CAS in the modulation of the Cyclic Electron Flow (CEF) could also be envisioned. CEF is recognized as a safety valve to minimize photo-oxidative damage (Pinnola and Bassi, 2018) and in the green alga *Chlamydomonas reinhardtii* CAS was shown to affect CEF under anoxia (Petroutsos et al., 2011). Finally, the phosphorylation status of CAS could be further altered by a so far unknown Ca^2+^-dependent kinase, whose activation might follow the physiological increases in stromal free Ca^2+^ levels regularly observed after the light-dark transition and in response to specific stress conditions (Sai and Johnson, 2002; Sello et al., 2016). It will thus be important in the future to properly assess how the overall phosphorylation status and the phosphorylation patterns of CAS are shaped at different daytimes and under different light and/or stress conditions. On the other hand, a more detailed assessment of the basal Ca^2+^ resting levels and stress induced Ca^2+^ transients in the stroma and thylakoid lumen of the *cas* mutant plants might also provide further suggestions for the role of CAS in shaping the plastidal Ca^2+^ landscape.

## Data Availability

All datasets for this study are included in the manuscript and/or the Supplementary Files.

## Author Contributions

EC, NP, and UV designed the experiments. UV conceived the project and supervised the experiments. EC performed most of the experiments. HR provided assistance in the bioinformatics and bioimaging analysis. NiP performed some of the Western blot analysis. VR performed the phosphoproteomics analysis. WW and MT supervised the phosphoproteomics analysis and were involved in the analysis of the data. VL performed the spectroscopic analysis together with EC and analyzed the data together with MG. UV and EC wrote the manuscript with contributions of all other authors.

## Conflict of Interest Statement

The authors declare that the research was conducted in the absence of any commercial or financial relationships that could be construed as a potential conflict of interest.

## Footnotes

1 http://www.maxquant.org

## Abbreviations

CAS: calcium sensor protein
CEF: Cyclic Electron Flow
GL: growth light
LHCII: light harvesting complex II (LHCII)
PGR5: proton gradient regulation 5
saGFP: self-assembly green fluorescent protein
SSU: small subunit of the Ribulose-1,5-bisphosphate carboxylase/oxygenase
STN: state transition kinase
TAP38: thylakoid-associated phosphatase 38.

## References

[B1] Arnon D. I. (1949). Copper enzymes in isolated chloroplasts. polyphenoloxidase in *Beta vulgaris*. *Plant Physiol.* 24 1–15. 10.1104/pp.24.1.1 16654194 PMC437905

[B2] Bellafiore S. Barneche F. Peltier G. Rochaix J. D. (2005). State transitions and light adaptation require chloroplast thylakoid protein kinase STN7. *Nature* 433 892–895. 10.1038/nature03286 15729347

[B3] Bhaskara G. B. Wen T. N. Nguyen T. T. Verslues P. E. (2017). Protein phosphatase 2Cs and microtubule-associated stress protein 1 control microtubule stability, plant growth, and drought response. *Plant Cell* 29 169–191. 10.1105/tpc.16.00847 28011693 PMC5304354

[B4] Bodenmiller B. Mueller L. N. Mueller M. Domon B. Aebersold R. (2007). Reproducible isolation of distinct, overlapping segments of the phosphoproteome. *Nat. Methods* 4 231–237. 10.1038/nmeth1005 17293869

[B5] Bonardi V. Pesaresi P. Becker T. Schleiff E. Wagner R. Pfannschmidt T. (2005). Photosystem II core phosphorylation and photosynthetic acclimation require two different protein kinases. *Nature* 437 1179–1182. 10.1038/nature04016 16237446

[B6] Bonaventura C. Myers J. (1969). Fluorescence and oxygen evolution from *Chlorella pyrenoidosa*. *Biochim. Biophys. Acta* 189 366–383. 10.1016/0005-2728(69)90168-6 5370012

[B7] Bonhomme L. Valot B. Tardieu F. Zivy M. (2012). Phosphoproteome dynamics upon changes in plant water status reveal early events associated with rapid growth adjustment in maize leaves. *Mol. Cell. Proteomics* 11 957–972. 10.1074/mcp.m111.015867 22787273 PMC3494150

[B8] Cabantous S. Terwilliger T. C. Waldo G. S. (2005). Protein tagging and detection with engineered self-assembling fragments of green fluorescent protein. *Nat. Biotechnol.* 23 102–107. 10.1038/nbt1044 15580262

[B9] Chen Y. Hoehenwarter W. Weckwerth W. (2010). Comparative analysis of phytohormone-responsive phosphoproteins in *Arabidopsis thaliana* using TiO2-phosphopeptide enrichment and mass accuracy precursor alignment. *Plant J.* 63 1–17. 10.1111/j.1365-313X.2010.04218.x 20374526

[B10] Cox J. Mann M. (2008). MaxQuant enables high peptide identification rates, individualized p.p.b.-range mass accuracies and proteome-wide protein quantification. *Nat. Biotechnol.* 26 1367–1372. 10.1038/nbt.1511 19029910

[B11] Cox J. Neuhauser N. Michalski A. Scheltema R. A. Olsen J. V. Mann M. (2011). Andromeda, a peptide search engine integrated into the MaxQuant environment. *J. Proteome Res.* 10 1794–1805. 10.1021/pr101065j 21254760

[B12] Datla R. S. Hammerlindl J. K. Panchuk B. Pelcher L. E. Keller W. (1992). Modified binary plant transformation vectors with the wild-type gene encoding NPTII. *Gene* 122 383–384. 10.1016/0378-1119(92)90232-e 1336757

[B13] Durek P. Schmidt R. Heazlewood J. L. Jones A. MacLean D. Nagel A. (2010). PhosPhAt, the *Arabidopsis thaliana* phosphorylation site database. An update. *Nucleic Acids Res.* 38 D828–D834. 10.1093/nar/gkp810 19880383 PMC2808987

[B14] Fan S. Meng Y. Song M. Pang C. Wei H. Liu J. (2014). Quantitative phosphoproteomics analysis of nitric oxide-responsive phosphoproteins in cotton leaf. *PLoS One* 9:e94261. 10.1371/journal.pone.0094261 24714030 PMC3979775

[B15] Fristedt R. Wasilewska W. Romanowska E. Vener A. V. (2012). Differential phosphorylation of thylakoid proteins in mesophyll and bundle sheath chloroplasts from maize plants grown under low or high light. *Proteomics* 12 2852–2861. 10.1002/pmic.201200196 22833285

[B16] Fristedt R. Willig A. Granath P. Crevecoeur M. Rochaix J. D. Vener A. V. (2009). Phosphorylation of photosystem II controls functional macroscopic folding of photosynthetic membranes in *Arabidopsis*. *Plant Cell* 21 3950–3964. 10.1105/tpc.109.069435 20028840 PMC2814517

[B17] Grieco M. Jain A. Ebersberger I. Teige M. (2016). An evolutionary view on thylakoid protein phosphorylation uncovers novel phosphorylation hotspots with potential functional implications. *J. Exp. Bot.* 67 3883–3896. 10.1093/jxb/erw164 27117338

[B18] Han S. Tang R. Anderson L. K. Woerner T. E. Pei Z. M. (2003). A cell surface receptor mediates extracellular Ca(2+) sensing in guard cells. *Nature* 425 196–200. 10.1038/nature01932 12968184

[B19] Heazlewood J. L. Durek P. Hummel J. Selbig J. Weckwerth W. Walther D. (2008). PhosPhAt, a database of phosphorylation sites in *Arabidopsis thaliana* and a plant-specific phosphorylation site predictor. *Nucleic Acids Res.* 36 D1015–D1021. 17984086 10.1093/nar/gkm812 PMC2238998

[B20] Hoehenwarter W. Thomas M. Nukarinen E. Egelhofer V. Rohrig H. Weckwerth W. (2013). Identification of novel in vivo MAP kinase substrates in *Arabidopsis thaliana* through use of tandem metal oxide affinity chromatography. *Mol. Cell. Proteomics* 12 369–380. 10.1074/mcp.M112.020560 23172892 PMC3567860

[B21] Ingelsson B. Vener A. V. (2012). Phosphoproteomics of *Arabidopsis* chloroplasts reveals involvement of the STN7 kinase in phosphorylation of nucleoid protein pTAC16. *FEBS Lett.* 586 1265–1271. 10.1016/j.febslet.2012.03.061 22616989

[B22] Kodru S. Malavath T. Devadasu E. Nellaepalli S. Stirbet A. Subramanyam R. (2015). The slow S to M rise of chlorophyll a fluorescence reflects transition from state 2 to state 1 in the green alga *Chlamydomonas reinhardtii*. *Photosynth. Res.* 125 219–231. 10.1007/s11120-015-0084-2 25663564

[B23] Koop H. U. Steinmuller K. Wagner H. Rossler C. Eibl C. Sacher L. (1996). Integration of foreign sequences into the tobacco plastome via polyethylene glycol-mediated protoplast transformation. *Planta* 199 193–201. 8680308 10.1007/BF00196559

[B24] Laemmli U. K. (1970). Cleavage of structural proteins during the assembly of the head of bacteriophage T4. *Nature* 227 680–685. 10.1038/227680a0 5432063

[B25] Larosa V. Meneghesso A. La Rocca N. Steinbeck J. Hippler M. Szabo I. (2018). Mitochondria affect photosynthetic electron transport and photosensitivity in a green Alga. *Plant Physiol.* 176 2305–2314. 10.1104/pp.17.01249 29284743 PMC5841685

[B26] Lenzoni G. Knight M. R. (2018). Increases in absolute temperature stimulate free calcium concentration elevations in the chloroplast. *Plant Cell Physiol.* 60 538–554. 10.1093/pcp/pcy227 30517735

[B27] Lin L. L. Hsu C. L. Hu C. W. Ko S. Y. Hsieh H. L. Huang H. C. (2015). Integrating phosphoproteomics and bioinformatics to study brassinosteroid-regulated phosphorylation dynamics in *Arabidopsis*. *BMC Genomics* 16:533. 10.1186/s12864-015-1753-4 26187819 PMC4506601

[B28] Lv D. W. Ge P. Zhang M. Cheng Z. W. Li X. H. Yan Y. M. (2014a). Integrative network analysis of the signaling cascades in seedling leaves of bread wheat by large-scale phosphoproteomic profiling. *J. Proteome Res.* 13 2381–2395. 10.1021/pr401184v 24679076

[B29] Lv D. W. Li X. Zhang M. Gu A. Q. Zhen S. M. Wang C. (2014b). Large-scale phosphoproteome analysis in seedling leaves of *Brachypodium distachyon* L. *BMC Genomics* 15:375. 10.1186/1471-2164-15-375 24885693 PMC4079959

[B30] Lv D. W. Subburaj S. Cao M. Yan X. Li X. Appels R. (2014c). Proteome and phosphoproteome characterization reveals new response and defense mechanisms of *Brachypodium distachyon* leaves under salt stress. *Mol. Cell. Proteomics* 13 632–652. 10.1074/mcp.M113.030171 24335353 PMC3916659

[B31] Machettira A. B. Gross L. E. Sommer M. S. Weis B. L. Englich G. Tripp J. (2011). The localization of Tic20 proteins in *Arabidopsis thaliana* is not restricted to the inner envelope membrane of chloroplasts. *Plant Mol. Biol.* 77 381–390. 10.1007/s11103-011-9818-5 21874592

[B32] Mehlmer N. Parvin N. Hurst C. H. Knight M. R. Teige M. Vothknecht U. C. (2012). A toolset of aequorin expression vectors for in planta studies of subcellular calcium concentrations in *Arabidopsis thaliana*. *J. Exp. Bot.* 63 1751–1761. 10.1093/jxb/err406 22213817 PMC3971373

[B33] Mekala N. R. Suorsa M. Rantala M. Aro E. M. Tikkanen M. (2015). Plants actively avoid state transitions upon changes in light intensity, role of light-harvesting complex II protein dephosphorylation in high light. *Plant Physiol.* 168 721–734. 10.1104/pp.15.00488 25902812 PMC4453798

[B34] Minagawa J. (2011). State transitions–the molecular remodeling of photosynthetic supercomplexes that controls energy flow in the chloroplast. *Biochim. Biophys. Acta* 1807 897–905. 10.1016/j.bbabio.2010.11.005 21108925

[B35] Murata N. (1969). Control of excitation transfer in photosynthesis. I. Light-induced change of chlorophyll a fluorescence in *Porphyridium cruentum*. *Biochim. Biophys. Acta* 172 242–251. 10.1016/0005-2728(69)90067-x 5775694

[B36] Nguyen T. H. Brechenmacher L. Aldrich J. T. Clauss T. R. Gritsenko M. A. Hixson K. K. (2012). Quantitative phosphoproteomic analysis of soybean root hairs inoculated with Bradyrhizobium japonicum. *Mol. Cell. Proteomics* 11 1140–1155. 10.1074/mcp.M112.018028 22843990 PMC3494206

[B37] Nomura H. Komori T. Kobori M. Nakahira Y. Shiina T. (2008). Evidence for chloroplast control of external Ca2+-induced cytosolic Ca2+ transients and stomatal closure. *Plant J.* 53 988–998. 10.1111/j.1365-313x.2007.03390.x 18088326

[B38] Nomura H. Komori T. Uemura S. Kanda Y. Shimotani K. Nakai K. (2012). Chloroplast-mediated activation of plant immune signalling in *Arabidopsis*. *Nat. Commun.* 3:926. 10.1038/ncomms1926 22735454

[B39] Olsen J. V. Blagoev B. Gnad F. Macek B. Kumar C. Mortensen P. (2006). Global, in vivo, and site-specific phosphorylation dynamics in signaling networks. *Cell* 127 635–648. 10.1016/j.cell.2006.09.026 17081983

[B40] Papageorgiou G. C. Govindjee (2011). Photosystem II fluorescence, Slow changes - Scaling from the past. *J. Photochem. Photobiol. B* 104 258–270. 10.1016/j.jphotobiol.2011.03.008 21530301

[B41] Pesaresi P. Hertle A. Pribil M. Schneider A. Kleine T. Leister D. (2010). Optimizing photosynthesis under fluctuating light, the role of the *Arabidopsis* STN7 kinase. *Plant Signal. Behav.* 5 21–25. 10.4161/psb.5.1.10198 20592803 PMC2835952

[B42] Pesaresi P. Pribil M. Wunder T. Leister D. (2011). Dynamics of reversible protein phosphorylation in thylakoids of flowering plants, the roles of STN7, STN8 and TAP38. *Biochim. Biophys. Acta* 1807 887–896. 10.1016/j.bbabio.2010.08.002 20728426

[B43] Petroutsos D. Busch A. Janssen I. Trompelt K. Bergner S. V. Weinl S. (2011). The chloroplast calcium sensor CAS is required for photoacclimation in *Chlamydomonas reinhardtii*. *Plant Cell* 23 2950–2963. 10.1105/tpc.111.087973 21856795 PMC3180803

[B44] Pinnola A. Bassi R. (2018). Molecular mechanisms involved in plant photoprotection. *Biochem. Soc. Trans.* 46 467–482. 10.1042/BST20170307 29666217

[B45] Pribil M. Pesaresi P. Hertle A. Barbato R. Leister D. (2010). Role of plastid protein phosphatase TAP38 in LHCII dephosphorylation and thylakoid electron flow. *PLoS Biol.* 8:e1000288. 10.1371/journal.pbio.1000288 20126264 PMC2811158

[B46] Reiland S. Finazzi G. Endler A. Willig A. Baerenfaller K. Grossmann J. (2011). Comparative phosphoproteome profiling reveals a function of the STN8 kinase in fine-tuning of cyclic electron flow (CEF). *Proc. Natl. Acad. Sci. U.S.A.* 108 12955–12960. 10.1073/pnas.1104734108 21768351 PMC3150903

[B47] Reiland S. Messerli G. Baerenfaller K. Gerrits B. Endler A. Grossmann J. (2009). Large-scale *Arabidopsis* phosphoproteome profiling reveals novel chloroplast kinase substrates and phosphorylation networks. *Plant Physiol.* 150 889–903. 10.1104/pp.109.138677 19376835 PMC2689975

[B48] Rintamaki E. Salonen M. Suoranta U. M. Carlberg I. Andersson B. Aro E. M. (1997). Phosphorylation of light-harvesting complex II and photosystem II core proteins shows different irradiance-dependent regulation in vivo. Application of phosphothreonine antibodies to analysis of thylakoid phosphoproteins. *J. Biol. Chem.* 272 30476–30482. 10.1074/jbc.272.48.30476 9374540

[B49] Rocha A. G. Mehlmer N. Stael S. Mair A. Parvin N. Chigri F. (2014). Phosphorylation of *Arabidopsis* transketolase at Ser(428) provides a potential paradigm for the metabolic control of chloroplast carbon metabolism. *Biochem. J.* 458 313–322. 10.1042/bj20130631 24328790 PMC3966265

[B50] Rochaix J. D. (2011). Regulation of photosynthetic electron transport. *Biochim. Biophys. Acta* 1807 375–383. 10.1016/j.bbabio.2010.11.010 21118674

[B51] Rochaix J. D. (2014). Regulation and dynamics of the light-harvesting system. *Annu. Rev. Plant Biol.* 65 287–309. 10.1146/annurev-arplant-050213-040226 24471838

[B52] Roitinger E. Hofer M. Kocher T. Pichler P. Novatchkova M. Yang J. (2015). Quantitative phosphoproteomics of the ataxia telangiectasia-mutated (ATM) and ataxia telangiectasia-mutated and rad3-related (ATR) dependent DNA damage response in *Arabidopsis thaliana*. *Mol. Cell. Proteomics* 14 556–571. 10.1074/mcp.M114.040352 25561503 PMC4349977

[B53] Rokka A. Aro E. M. Vener A. V. (2011). Thylakoid phosphoproteins: identification of phosphorylation sites. *Methods Mol. Biol.* 684 171–186. 10.1007/978-1-60761-925-3_15 20960130

[B54] Rose C. M. Venkateshwaran M. Volkening J. D. Grimsrud P. A. Maeda J. Bailey D. J. (2012). Rapid phosphoproteomic and transcriptomic changes in the rhizobia-legume symbiosis. *Mol. Cell. Proteomics* 11 724–744. 10.1074/mcp.M112.019208 22683509 PMC3434772

[B55] Roustan V. Bakhtiari S. Roustan P.-J. Weckwerth W. (2017). Quantitative in vivo phosphoproteomics reveals reversible signaling processes during nitrogen starvation and recovery in the biofuel model organism *Chlamydomonas reinhardtii*. *Biotech. Biofuels* 10:280. 10.1186/s13068-017-0949-z 29209414 PMC5704542

[B56] Ruban A. V. Johnson M. P. (2009). Dynamics of higher plant photosystem cross-section associated with state transitions. *Photosynth. Res.* 99 173–183. 10.1007/s11120-008-9387-x 19037743

[B57] Ruge H. Flosdorff S. Ebersberger I. Chigri F. Vothknecht U. C. (2016). The calmodulin-like proteins AtCML4 and AtCML5 are single-pass membrane proteins targeted to the endomembrane system by an N-terminal signal anchor sequence. *J. Exp. Bot.* 67 3985–3996. 10.1093/jxb/erw101 27029353 PMC4915527

[B58] Sai J. Johnson C. H. (2002). Dark-stimulated calcium ion fluxes in the chloroplast stroma and cytosol. *Plant Cell.* 14 1279–1291. 10.1105/tpc.000653 12084827 PMC150780

[B59] Schönberg A. Rodiger A. Mehwald W. Galonska J. Christ G. Helm S. (2017). Identification of STN7/STN8 kinase targets reveals connections between electron transport, metabolism and gene expression. *Plant J.* 90 1176–1186. 10.1111/tpj.13536 28295753

[B60] Seigneurin-Berny D. Salvi D. Dorne A. J. Joyard J. Rolland N. (2008). Percoll-purified and photosynthetically active chloroplasts from *Arabidopsis thaliana* leaves. *Plant Physiol. Biochem.* 46 951–955. 10.1016/j.plaphy.2008.06.009 18707896

[B61] Sello S. Perotto J. Carraretto L. Szabo I. Vothknecht U. C. Navazio L. (2016). Dissecting stimulus-specific Ca2+ signals in amyloplasts and chloroplasts of *Arabidopsis thaliana* cell suspension cultures. *J. Exp. Bot.* 67 3965–3974. 10.1093/jxb/erw038 26893493 PMC4915524

[B62] Shapiguzov A. Ingelsson B. Samol I. Andres C. Kessler F. Rochaix J. D. (2010). The PPH1 phosphatase is specifically involved in LHCII dephosphorylation and state transitions in *Arabidopsis*. *Proc. Natl. Acad. Sci. U.S.A.* 107 4782–4787. 10.1073/pnas.0913810107 20176943 PMC2842063

[B63] Silverstein T. Cheng L. Allen J. F. (1993). Chloroplast thylakoid protein phosphatase reactions are redox-independent and kinetically heterogeneous. *FEBS Lett.* 334 101–105. 10.1016/0014-5793(93)81690-2 8224208

[B64] Stael S. Rocha A. G. Wimberger T. Anrather D. Vothknecht U. C. Teige M. (2012). Cross-talk between calcium signalling and protein phosphorylation at the thylakoid. *J. Exp. Bot.* 63 1725–1733. 10.1093/jxb/err403 22197893 PMC3970089

[B65] Thompson J. D. Gibson T. J. Plewniak F. Jeanmougin F. Higgins D. G. (1997). The CLUSTAL_X windows interface, flexible strategies for multiple sequence alignment aided by quality analysis tools. *Nucleic Acids Res.* 25 4876–4882. 10.1093/nar/25.24.4876 9396791 PMC147148

[B66] Tikkanen M. Aro E. M. (2012). Thylakoid protein phosphorylation in dynamic regulation of photosystem II in higher plants. *Biochim. Biophys. Acta* 1817 232–238. 10.1016/j.bbabio.2011.05.005 21605541

[B67] Tikkanen M. Grieco M. Kangasjarvi S. Aro E. M. (2010). Thylakoid protein phosphorylation in higher plant chloroplasts optimizes electron transfer under fluctuating light. *Plant Physiol.* 152 723–735. 10.1104/pp.109.150250 19965965 PMC2815896

[B68] Tikkanen M. Piippo M. Suorsa M. Sirpio S. Mulo P. Vainonen J. (2006). State transitions revisited-a buffering system for dynamic low light acclimation of *Arabidopsis*. *Plant Mol. Biol.* 62 779–793. 10.1007/s11103-006-9044-8 16897465

[B69] Tyanova S. Temu T. Sinitcyn P. Carlson A. Hein M. Y. Geiger T. (2016). The perseus computational platform for comprehensive analysis of (prote)omics data. *Nat. Methods* 13 731–740. 10.1038/nmeth.3901 27348712

[B70] Vainonen J. P. Hansson M. Vener A. V. (2005). STN8 protein kinase in *Arabidopsis thaliana* is specific in phosphorylation of photosystem II core proteins. *J. Biol. Chem.* 280 33679–33686. 10.1074/jbc.m505729200 16040609

[B71] Vainonen J. P. Sakuragi Y. Stael S. Tikkanen M. Allahverdiyeva Y. Paakkarinen V. (2008). Light regulation of CaS, a novel phosphoprotein in the thylakoid membrane of *Arabidopsis thaliana*. *FEBS J.* 275 1767–1777. 10.1111/j.1742-4658.2008.06335.x 18331354

[B72] Vener A. V. Strålfors P. (2005). Vectorial proteomics. *IUBMB Life* 57 433–440. 10.1080/15216540500138360 16012052

[B73] Vener A. V. van Kan P. J. Rich P. R. Ohad I. Andersson B. (1997). Plastoquinol at the quinol oxidation site of reduced cytochrome bf mediates signal transduction between light and protein phosphorylation: thylakoid protein kinase deactivation by a single-turnover flash. *Proc. Natl. Acad. Sci. U.S.A.* 94 1585–1590. 10.1073/pnas.94.4.1585 11038603 PMC19835

[B74] Voinnet O. Rivas S. Mestre P. Baulcombe D. (2003). An enhanced transient expression system in plants based on suppression of gene silencing by the p19 protein of tomato bushy stunt virus. *Plant J.* 33 949–956. 10.1046/j.1365-313x.2003.01676.x 12609035

[B75] Wang H. Gau B. Slade W. O. Juergens M. Li P. Hicks L. M. (2014). The global phosphoproteome of *Chlamydomonas reinhardtii* reveals complex organellar phosphorylation in the flagella and thylakoid membrane. *Mol. Cell. Proteomics* 13 2337–2353. 10.1074/mcp.M114.038281 24917610 PMC4159653

[B76] Wang L. Yamano T. Takane S. Niikawa Y. Toyokawa C. Ozawa S.-I. (2016). Chloroplast-mediated regulation of CO2-concentrating mechanism by Ca2+-binding protein CAS in the green alga *Chlamydomonas reinhardtii*. *Proc. Natl. Acad. Sci. U.S.A.* 113 12586–12591. 10.1073/pnas.1606519113 27791081 PMC5098658

[B77] Wang X. Bian Y. Y. Cheng K. Gu L. F. Ye M. L. Zou H. F. (2013). A large-scale protein phosphorylation analysis reveals novel phosphorylation motifs and phosphoregulatory networks in *Arabidopsis*. *J Proteomics* 78 486–498. 10.1016/j.jprot.2012.10.018 23111157

[B78] Weinl S. Held K. Schlucking K. Steinhorst L. Kuhlgert S. Hippler M. (2008). A plastid protein crucial for Ca2+-regulated stomatal responses. *New Phytol.* 179 675–686. 10.1111/j.1469-8137.2008.02492.x 18507772

[B79] Whiteman S. A. Nuhse T. S. Ashford D. A. Sanders D. Maathuis F. J. (2008). A proteomic and phosphoproteomic analysis of *Oryza sativa* plasma membrane and vacuolar membrane. *Plant J.* 56 146–156. 10.1111/j.1365-313X.2008.03578.x 18557835

[B80] Wunder T. Xu W. Liu Q. Wanner G. Leister D. Pribil M. (2013). The major thylakoid protein kinases STN7 and STN8 revisited, effects of altered STN8 levels and regulatory specificities of the STN kinases. *Front. Plant Sci.* 4:417. 10.3389/fpls.2013.00417 24151498 PMC3801152

[B81] Yang Z. Guo G. Zhang M. Liu C. Y. Hu Q. Lam H. (2013). Stable isotope metabolic labeling-based quantitative phosphoproteomic analysis of *Arabidopsis* mutants reveals ethylene-regulated time-dependent phosphoproteins and putative substrates of constitutive triple response 1 kinase. *Mol. Cell. Proteomics* 12 3559–3582. 10.1074/mcp.M113.031633 24043427 PMC3861708

[B82] Zheng L. Baumann U. Reymond J. L. (2004). An efficient one-step site-directed and site-saturation mutagenesis protocol. *Nucleic Acids Res.* 32:e115. 10.1093/nar/gnh110 15304544 PMC514394

