# Supplementary material for: The High Light Response in Arabidopsis Requires the Calcium Sensor Protein CAS, a Target of STN7- and STN8-Mediated Phosphorylation
Source: Front Plant Sci. 2019 Jul 30;10:974. doi: 10.3389/fpls.2019.00974 (PMC6682602; doi:10.3389/fpls.2019.00974)
Supplement: Supplementary file 1 [file Data_Sheet_1.PDF]

Supplementary Data S1: Sequence alignment of CAS proteins from dicots

```
                                                                                             :  ::                    *: :: ::   *. .      .            :**
S_indicum          --------MALRASS-------AVRPPSLQLSPPQ------------TPP--KSVILP---PKVN-------SLSLPATAILPLFALFAA----PHEARAE-ILPKEQIVSSLTQVESAIDQLQELGSNFFSTASQVVGAVADAVKP-- 150
E_guttata          -------MMALRASS-------AARPPSVSQSPPL-------------LSPSPKTVLLPSKAPPVN-------SLSLPAASILPLFALFAA----PIEARAQ-IFPKEQIVSSITQVESTIDQIQELGSSFFSSAGQVIGAVTNAVKPGV 150
C_canephora        --------MALRASS-------ATARP-PLPSPPPL--------PSS-SSPSKTFVPITKIPPSSNRLKSNPLVVSLPTSTALSILTLFSAP---SYEARAI-SLPKEQIVSSLDQLESTIDQVQDVGSSIFDVAGKVIGAAVEVVKPGI 150
I_nil              -------MMAVRAPA-------TAPPP-PLPLPP---------------SPRLKASSLSKLPPKPE-FSPNSLSVSLTTSTTLSLLPLLTAP---SYEAKAL-SFPKEEIVSSLTLVESTINQVQQLGLSAFGNAGRVIGPVVEFVKPGI 150
N_tabacum          --------MALRASA-------TAKS--PLPPP-----------SSS----PTKIFNFPTLSQKPE-FTSKSVSLSLSTSTALFLFPLFTA----THEARAL-SLPKEDIVSSLNQVESAVNQAQQVGSNIFDAASRVIGPVIEFVKPGI 150
N_tomentosiformis  --------MALRASA-------TAKS--PLPPP-----------SSS----PTKIFNFPALSQKPE-FTSKSVSLSLSTSTALFLFPLFTA----THEARAL-SLPKEDIVSSLNQVESAVNQAQQVGSNIFDAASRVIGPVIEFVKPGI 150
N_attenuata        --------MALRASA-------TVKS--PLPPA-----------SSSSSSPTKIFNFPTLSQKPE-FTSKSVSVSLSTSTALFLFPLFTA----THEARAL-SLPKEDIVSSLNQVESAVNQAQEVGSNIFDAASRVIGPVIEFVKPGI 150
N_benthamiana      --------MALRASA-------TVKS--HLPPP-----------SSS----PIKIFNFPTLSQKPE-FTSNSVSVSLSTSTALFLFPLFTA----THEARAL-SLPKEDIVSSLNQVESAVNQAQEVGSNIFDAASRVIGPVIEFVKPGI 150
N_sylvestris       --------MALRASA-------TVKS--PLPPP-----------SSSSSTPTKIFNFPTLSQKPE-FTSKSVSVSLSTSTALFLFPLFTA----THEARAL-SLPKEDIVSSLNQVESAVNQAQQVGSNIFDAASRVIGPIIEFVKPGV 150
S_pennellii        --------MAIRASA-------TAKSPLPLPPPPPP---------SSS-TSSPPKVFSLPKLTQKL-------VSVSFSTSTALFLFPLFTA----THEARAI-TLPKEDIVSSLNQVESVVNQAQEVGSNIFDTVSGVIGPVIEFVKPGI 150
S_lycopersicum     --------MALRASA-------TAKSPLPLPPPPPS---------SSSSTSSPPKVFSLPKLTQKP-------VSVSFSTSTALFLFPLFTA----THEARAI-NLPKEDIVSSLNQVESVVNQAQEVGSNIFDTVSGVIGPVIEFVKPGI 150
S_tuberosum        --------MALRASA-------TAKS--PLPPPPSS---------SSSSTSSPPIVLNLPKLTQKT-------VSVAFSTSTALFLFPLFTA----THEARAI-SLPKEDIVSSLNQVESAVNQAQEVGSNIFDTISGVIGPVIEFVKPGI 150
C_annuum           --------MALRASA-------TTKS--PLPPPPSS---------SSSSSSVPKQFSLPKLSQKPE-FTTKSLSVSFSTSTALFLFPLLTA----THEARAI-SLPKEDIVSSLNQVESAVNQAQEVGSNIFDTASKVIGPVIDFVKPGI 150
H_annuus           --------MALRASA-------TASLNHHSPPPP---------------SP-KYLIPFKPTPKIRP---DSSSLSLSTSTTLSLLALFTA----PIEANAL-AISKDDIVSSITQVEEKIDQVQKVGSDVFELTGNVIKTVSDFLKPAM 150
C_cardunculus      --------MALRAST-------TARLRHHQPPPP---------------PPPKTLIPFKPTPRIQPR--PDSYLSLSTSTTLSLLTLFTS----PIEAHAF-TISKDDVVSSLTQVEEKIDLVQKVGYNVFEFTGNAVKTVTDFLKPAA 150
D_carota           --------MALRASA-------TAR-----PPIS---------------SPPRTSLPAKTTQRPQVF--KPKSVSVSTTNTLSLLALFTA----PLDAKAI-SLPKEQIVSSITQVEDTIGQVQEVGSNVFDVLGKVFGAVSQAVKPGV 150
S_superba          ------MAMALRASA-------TARP--PLPPPP------------SSPFPPPRTLSLSKSSSKSQ---FRPYSASLPISTTLSLFALFTT----PHEARAL-SLSKEQIVSSLTQVEKTIDQVQEAGSSFFDVAQRVIRVVAETVKPGI 150
A_thaliana         ------MAMAEMATK-------SSLS-AKLTLPS--------------SST----KKTLSLRQVS---------VSLPTSTSISLLSLFASP---PHEAKAAVSIPKDQIVSSLTEVEKTINQVQETGSSVFDATQRVFQVVGDALKPAL 150
A_lyrata           ------MAMAEMAMK-------SSLS-AKLTLPS--------------SSS----KK--TLRQIS---------VSLPTSTSISLLSLFASP---PHEAKAAVSISKDQIVSSITEVEKTINQVQETGSSVFDATQRVFQVVGDALKPAL 150
C_sativa           ------MAMAEMAMK-------SSVS-AKLTLPS--------------SSS-TF-CKKTAVRQIS---------VSLPTSTSISLLSLFASP---PHEAKAAVSISKDQIVSSLTEVEKTINQVQETGSSVFDATQRVFQVVGDALKPAL 150
C_rubella          ------MAMAEMAMK-------SSVS-AKLTLPS--------------SSSSSF-CKKT-VRQIS---------VSLPTSTSISLLSLFASP---PHEAKAAVSISKDQIVSSLTEVEKTINQVQETGSSVFDATQRVFQVVGDALKPAL 150
B_rapa             ------MAMAEMAMK-------SSVTTAKLTLPP--------------SSSS---CKKR-VRQVS---------VALPTTTSISLLSVFSSP---PPEAKAAVSISKDQIVSSLTEVEKTINQVQETGSSVFDATQRVFQVVGDALKPAL 150
B_napus            ------MAMAEMAMK-------SSVTTAKLTLPP--------------SSSS---CKKR-VRQVS---------VALPTTTSISLLSVFSSP---PPEAKAAVSISKDQIVSSLTEVEKTINQVQETGSSVFDATQRVFQVVGDALKPAL 150
B_oleracea         ------MAMAEMAMK-------SSVTTTKLTLPP--------------SSSSS--CKKR-ARQVS---------VALPTTTSISLLSVFSSP---PPEAKAAVSISKDQIVSSLTEVEKTINQVQETGSSVFDATQRVFQVVGDALKPAL 150
R_sativus          ------MAMAEMAMK-------SSVT-ANLTIPP--------------SSSSSS-CKKR-VRQIS---------VALPTTTSISLLSVFSSP---PPEAKAAVSISKDQIVSSLTEVEKTINQVQETGSSVFDATQRVFQVVGDALKPAL 150
E_salsugineum      ------MAMAEMAMK-------SSVTAKLTILSS--------------SSSSSS-CKKR-ERQIS---------VALPTSTSISLLSLFASP---PHEAKAAVSIAKDQIVSSLTEVEKTINQVQETGSSVFDATQRVFQVVGDALKPAL 150
N_caerulescens     ------MAMAEMAMK-------SSVT-AKLTLPS--------------SSSFSSSCKKRSVRQIS---------VALP--TSISLLSLFASP---PHEAKAAVSISKDQIVSSITEVEKTINQVQETGSSVFDATQRVFQLVGDALKPAF 150
B_vulgaris         ------MAMEMALRA-------SASS-SKPTLP-----------------SSSSQKTLFSKQNSKFQLKPAQVQLFSTSTSLSLLTLFTTP------EAKAVALPKEQLVTSLTQVEQTLDQVQQVGSDVFDSAQKVFQVVAGALKPGI 150
S_oleracea         ------MAMEMMALR-------VSAS-AKPTISP--------------SSSSSSSQKSQLLKQSSKLQLKQAQNLFFPTSTSLSLLTLFTTP---F--DAKAVSLPKEQLVTSLTQVEQTIDQVQEVGSSVFDSAQKVFQVVAEALKPGI 150
P_mume             ------MAVEMVIRA-------SATPRPSLPP----------SPSPSLSATTKCSLKP-KFRPTSVS---------LPTST-TISLLALFT-S---PFEAKAL-SLSKDQIVSSLTEVEKTIDQVQQVGSTVFDGTERVLEAILNAVKPGI 150
P_persica          ------MALEMAIRA-------SATPRPSLPP----------SPSPSLSATTKCSLKP-KFRPTSVS---------LPTS--TISLCALFT-S---PFEAQAF-SLSKDQIVSSLTEVEQTIDQVQQAGSTVFEGTERVLEAILNAVKPGI 150
M_domestica        ------MAVEMAIRA-------SATPRPSLP-----------QPSP---ATPKLSLKPPKFRPTSVS---------LPTST-TISLLALFT-S---PIEAKAF-SLSKDQIVSSLTEVEQTIDQIQQVGSTVFDGTAHVLEAVLNAVKPGI 150
F_vesca            ------MAVEMAIRA-------SVTPRPHLPPPS-----SSPSPSSSSPSIPKFSFKP-QFRPVSVS---------LPTST-TLSLLALFT-S---PFEAKAF-SLSKDQIVSSLTEVEQTIDQVQQVGSSVFDNTGRFLESFLNAVKPGI 150
Z_jujuba           ------MAMEMAIRA-------SAVQRPPQSP------------PSRSSTPRATFKP-QLRATSVS---------LPAST-TISLLAIFT-P---LNEAKAL-SLSKDQIVSSLDEVEKTIDQVQQVGSIFFDAAQRVLEVVGNALKPGL 150
C_chinensis        ------MAMELAIRA-------SATPRLSRPT----------ISSLSSTPR--ASSRP-QFRPTSVS---------LPTST-PISLLALFT-A---PHEAKAL-SLSKDQIVSSITEAEKAIDQVQEVGSSFFDTTKRVLEGVGNVLKPGI 150
J_regia            ------MAMELAIRA-------SATPRPSLPS----------SSSLSSKPKPIPSSRP-HFRPISVS---------LPTST-TISLLALFT-P---PREAKAL-SLPKDQIVSSLTEVEKTIEKVQEVGSSFLDITKQIFEVVRNALKPGI 150
P_trichocarpa      ------MAMEMAIRS-------SFTTRLS-------------PRSTSSTTKPSFFKPHQLRPISVS---------LPTS--TISLLSLFA-P---PNEAKAL-TISKDQVVSTLTDVEKTIDQAVEFSSSVLDSAQKVFEGVTNSLKPGI 150
P_euphratica       ------MAMEMAIRS-------SFTARLS-------------PRSTSSTTKPSFFIPHQLRPISVS---------LPTSTLTISLLSLFA-P---PNEAKAL-TISKDQVVSTLTDVEKTIDQVVEFSSSVLDSAQKVVEGVTNSLKPGI 150
C_follicularis     ------MAVEIAITA-------SRNARLSSS--------VAPPPSLSSTTKSS---KALFRPSIVS---------LPIST-TISLLSLFT-P---PLEAKALSTLSKDQIVSSITEVEKTIDQVQEVGSSFLDTTQRVLEVVGNALKPAI 150
L_formosana        ------MAMEMASEL-------QQLLGLLSLLFS-----LSCTVRASSPLPKHT--SKPQFRPTSVS---------LPTS-VS--LFALFA-A---PHEAKAL-SLSKDQIVSSLTGVEKTIDQVQVVGSGLFDSTQRVFQVVANALKPGI 150
V_vinifera         ------MALQASATV-------RHPFSAPPP--S-----LSPTPRASSSPFKPP--PKPQLRPISIS---------LPAS-ITIPLFSLFT-P---PHEAKAV-SIPKDQIVSSITEVEKKIDEIQEVGSGFLDTTQRILEVVANALKPGI 150
C_capsularis       ----MAAAAQMAIRV-------SASARLPLPPPPSSSSSPSPSLAPPSTTTSKPSWKT-QLKPISLI---------LPTST-TLSLLALFA-P---PHDAKAA-TLTKDQIVSSLNEVEKTIDQVQEASSSALATAQQILDVLGKALKPAI 150
C_olitorius        MAAPAAAAAQMAIRV-------SASARLPLPPPPSSSS-PSPSVAPPSATTTKPCFKT-QLKPISLI---------LPTST-TLSLLALFS-P---PHDAKAA-TLTKDQIVSSLNEVEKTIDQVQEASSSALATAQQILDVLGKALKPAI 150
G_hirsutum         ------MAVQMAIRV-------SPTARLSLPPP--SPSPPSPSV-PSSTISTKPFLKPQQPKPISLS---------LPTST-AISLLALFS-P---PHEAKAI-NLSKEQIISSLNEVEKTIDQVQEASSSALDVAQQIFDVVGKVLKPAI 150
G_arboreum         ------MAVQMAIRV-------SPTARLSLPPP--SPSPPSPSV-PSSAISTKPFLKPQQPKPISLS---------LPTST-AISLLALFS-P---PHEAKAI-ILSKEQIISSLNEVEKTIDQVQEASSSALDVAQQIFDVVGKVLKPAI 150
G_raimondii        ------MAVQMAIRV-------SPTARLSLPPP--SPSPPSPSV-PSSIISTKPSLKPRQPKPISLS---------LPTST-AISLLALFS-P---PHEAKAI-NLSKEQIISSLNEVEKTIDQVQEASSSALDVAQQIFDVVGKVLKPAI 150
T_cacao            ------MAVQMTVRV-------SATARLSLPPL--SSSPSSPSP-SGASSTSKVSLKP-QFKPISLS---------LPTST-AISLLALFS-P---PHEAKAV-SLSKEQIVSSLTEVEKTLNQVQEAGSTAFDTVQQFLDIVGKALKPAI 150
C_sinensis         ------MAVVMAIRS-------SATAKLSVPAS------------PSVASAPKSYSSKAQLKPNSVL---------LPTST-SFSLLALFN-A---PNEVKAL-TLDKDQIVSSLNEVEKTIDQVQEAGSSFLETTQKVLDAVGSALKPAV 150
J_curcas           ------MAAEMALRI---RSSSTATARLSLPSSS-----------STS-SSSPS----KALRPISVS---------LPTST-TISFLAVFSSP---PQEAKAL-SLSKDQIVSSLNDVEKTIDQVQEVGSGFLDTAQRVFGIVSDALKPGI 150
M_esculenta        ------MAAQMALTS---PSSCSATSRLSLPLSP-----------STS-SPSKSSFFKPLLKPISVS---------LPTSI-TISFLALFT-P---PHEAKAL-TLSKDQIVSSLNEVEKTIDQVQEVGSDFFDTAQRVFGIVSDALKPGV 150
R_communis         ---MALAAEKMALRSSTASSSSSATAKLSLPSSP-----------STSNSPSKPSFFKPQLRPISVS---------LPTST-TISLLAVFT-P---PFEAKAL-P--KDQIVSSLTEVEKTIDQVQEVGSGILDTAQRVFGIVSDALKPGI 150
T_subterraneum     ----MAMEITAMASL-------PTTTRASLPTPST-----------SPHTITTF---SKPQFKRIS---------LPTST-TISLLSLFT-I---PNEARAAVNISKDQIVSSITQVEQTIDQVQEVGSGFLDSAQRVAEAIGNVLKPGI 150
M_truncatula       ----MAMEIAAMASL-------PTP-KPSLPSPST-----------LPHTITTF---SKPQLRRIS---------LPTST-TISLLTLFI-P---PNEARAAVNISKDQIVSSLTQVEQTIDQVQVVGSGFLDSAQRVAEAVGSALKPGF 150
C_arietinum        ----MALEITAMASS-------TTR--PSLPTPST-----------T-FSKPLF---KRLHNINIS---------LPTST-TISLLALFT-P---PNEARAAVNISKDQIVSSLTQVEQTIDQVQEVGSGFLDSAQRVVEAIGNVLKPGF 150
L_angustifolius    ------MEISAMAST-------TPS-----STPSL-----------TLNTFCKP---PQLKHSHIS---------LPTST-TISLLTLFS-P---PYEAKA---FSKDQILSSITQVEKTIDEVQDVGSSFLDTSQHVFEAIGNALKPGI 150
G_max              ------MEISALASS-------ATP-RPSLTTPST-----------SPHTVVTTTFSKPQLRRSHVAV-----SLPTST-TISLLALFA-P---PSEAKAAVSIAKDQIVSSLTQVEKTLDQVQEVGSGVLDTAQRIAEVIGNALKPGI 150
G_soja             ------MEITAMASS-------ATPPRPSLPTPST-----------SPHTVVTTTFSKPQLRRSHIA--------LPTST-TISLLALFA-P---PNEAKAAVSIAKDQIVSSLTQVEKTLDQVQEMGSGVLDTAQRVAEVIGNALKPGI 150
V_angularis        ------MEISAMASS-------ATP-RPSLPTP-------------SSHPVVTT-FTKPQLRRNHIA--------LPTSS-TISLLALFA-P---PNEAKAAVSIAKDQIVSSLTQVEKTIDQVQEVGSNVLDTAQRVAEVIGNALKPGI 150
V_radiata          ------MEISAMASS-------ATP-RPSLPTP-------------SPHPVVTT-FTKPQLRRNHIA--------LPTSS-TISLLALFA-P---PNEAKAAVSIAKDQIVSSLTQVEKTIDQVQEVGSSVLDTAQRVAEVIGNALKPGI 150
P_vulgaris         ------MEIAAMASS-------VTP-RPSLPTP-------------SPQTVVTT-FSKPQLRRTQIA--------LPTAS-TISILALFA-P---PNEAKAAVSIAKDQIVSSLTQVEKTIDQVQEVGSSVLDTAQRVAEVIGNALKPGI 150
C_cajan            ------MEITTMASS-------ATP-----RPP-------------SPHAVVTT-FNKPQPRRVHVA--------LPTST-TISLLALFA-P---PNEAKA---IAKDQIVSSLTQVEKTIDQVQEVGSSVFDTAQRVAEVIGNALKPGI 150
A_duranensis       ------MEISAIMAS-------ATP-RPSLPIPTPSSS---SSSPSSSTLVSNSKLPLLKRSSHIT--------LPTST-TISLLALFS-P---PHEAKAALTIPKDQIVSSLNDVEKTIDQVQVVGSSFLDAAQRVLEIVGNALKPGI 150
A_ipaensis         ------MEISAIMAS-------ATP-RPSLPIPTPSSS---SSS-SSSTLVSNSKLPLLKRSSHIT--------LPTST-TISLLALFS-P---PHEAKAALTIPKDQIVSSLNDVEKTIDQVQVVGSSFLDAAQRVLEIVGNALKPGI 150
C_melo             ----MAMKIPIRASS-------IPR--HHHPTPSLP----------SPSHRSDS---KSQFRPISVS---------LPAST-TLSLLALIS-S---PYEARA---LNKDQIVSSLNEVEKTFDQVQEMGSNFFDIAQQAIESAKNVLKPGV 150
C_sativus          ----MAMHIHIRASS-------IPR--HHHPTPSLP----------SPSHRSDS---KSQFRPISVS---------LPAST-TLSLLALFS-S---PYEAKA---LNKDQIVSSLNEVEKTFDQVQEMGSNFFDIAQQAIESAKTVLKPGV 150
N_nucifera         ------MEMAFRAAA-------TARPPPSSSSSVLP---------FAPRASLSKPTPRTQVRPASVS---------LPTTSAAISLLALFT-A---PNDAKAF-SFSKEQIVSTLTQVENTVDQVQEVSSGVLEFSQRVLQVVIDTLKPGV 150
E_guineensis       ------MAMAFRAAV-------VTRPPPP------------RKPASKSQPGP---------SPPLL---------SPTPTALSLLALFSTP-SSHCEAKAF-AFSKDGIINSLEKVENAIDQVEDVGSRVLDFSQNVIKVLVDALKPGV 150
P_dactylifera      ------MAMAFRAAA-------VTRPPPP------------RKPAPKSQPGP---------SPPLL---------SPTPTALSLLALFSAP-SSHCEAKAF-DFSKDDILNSLEKVENAIDRVGDVGFRVWDLSRSVIEVLTDALKPGV 150
A_comosus          --------MAFRAAA-------VARPPPPPPSPS--------SSSSSSTKPNPRALRRASSSSPPRS---------HASPAALSLVALFATS-ASYSEAKAL-AFSKESIVDSLTKVESTIDQVEDVGSKVVDFTLNIFKVLSDTLKPGV 150
A_officinalis      ------MALAFRATA-------AIRIRPP------------PQSKPKPKPNKHSLLLPPPPPPPPQNNEQHSLVSSTSTTAISFLALLSIP-NGFCEAKAF-AFSKEDIVSSLTKVEDTIDQVGGAGSKALDFSQQVIKVLGDTLKPAV 150
D_catenatum        ------MAMAVRPAA-------VRRPPHL------------PKYALKPQPRIS--------HPLLL---------CPSPAALSLLALFSQPQTSFLEAKAF-TLPKEGIVSSLTKVEETIDQVGEAGSKALDFSKDIVKVVTDSLKPIV 150
P_equestris        ------MAMVVRAAG-------VSRPPCL------------PKSALKPKPRN---------TPFML---------SPSPAALSLLVLFAQPHTSF-EAKAF-SLPKEDIVSSLTKVEETIDQAAEVGSKALEFSREIFKGVIDAVKPAV 150
```

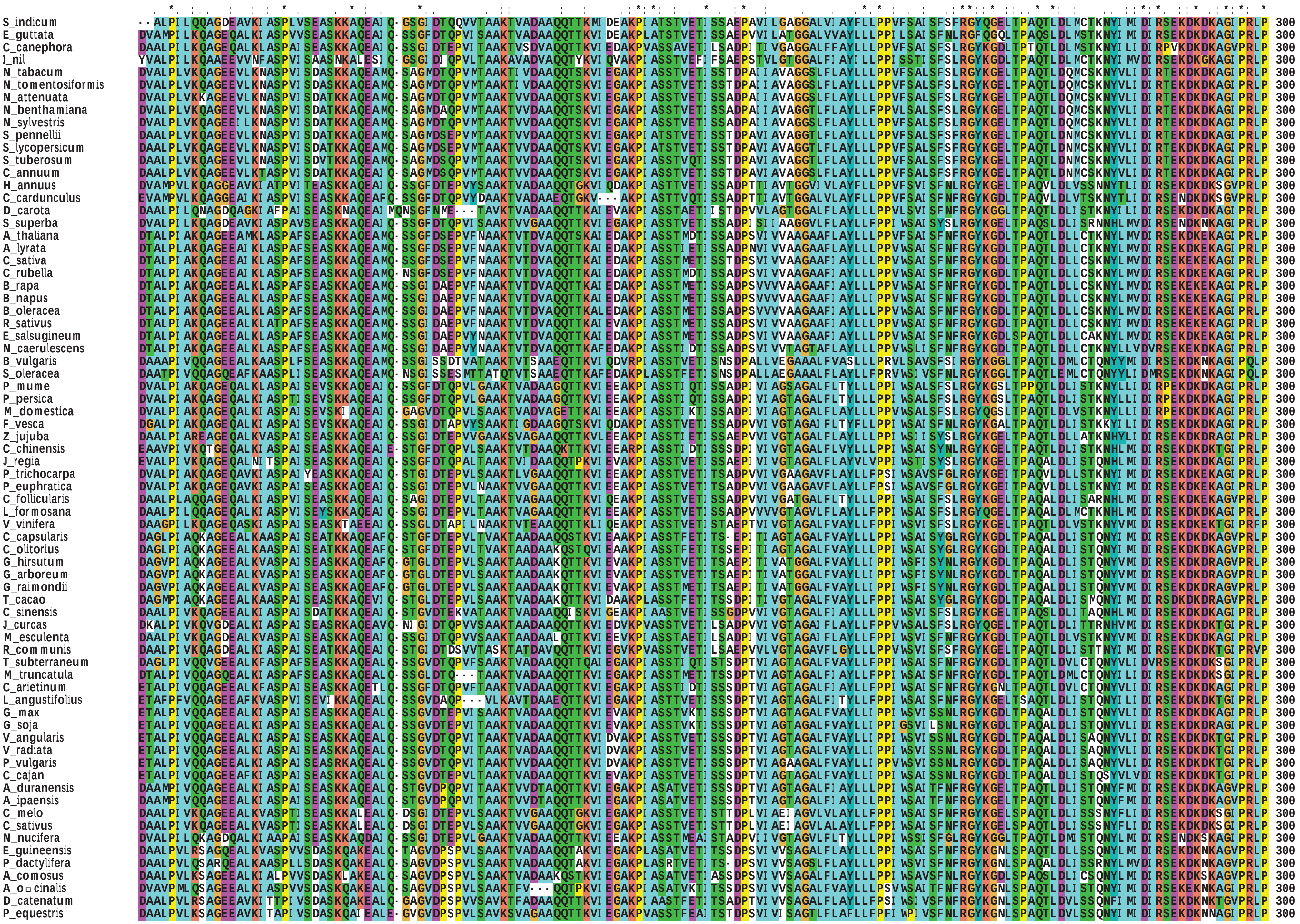
S_indicum
E_guttata
C_canephora
I_nil
N_tabacum
N_tomentosiformis
N_attenuata
N_benthamiana
N_sylvestris
S_pennellii
S_lycopersicum
S_tuberosum
C_annuum
H_annuus
C_cardunculus
D_carota
S_superba
A_thaliana
A_lyrata
C_sativa
C_rubella
B_rapa
B_napus
B_oleracea
R_sativus
E_salsugineum
N_caerulescens
B_vulgaris
S_oleracea
P_mume
P_persica
M_domestica
F_vesca
Z_jujuba
C_chinensis
J_regia
P_trichocarpa
P_euphratica
C_follicularis
L_formosana
V_vinifera
C_capsularis
C_olitorius
G_hirsutum
G_arboreum
G_raimondii
T_cacao
C_sinensis
J_curcas
M_esculenta
R_communis
T_subterraneum
M_truncatula
C_arietinum
L_angustifolius
G_max
G_soja
V_angularis
V_radiata
P_vulgaris
C_cajan
A_duranensis
A_ipaensis
C_melo
C_sativus
N_nucifera
E_guineensis
P_dactylifera
A_comosus
D_catenatum
P_equestris
300

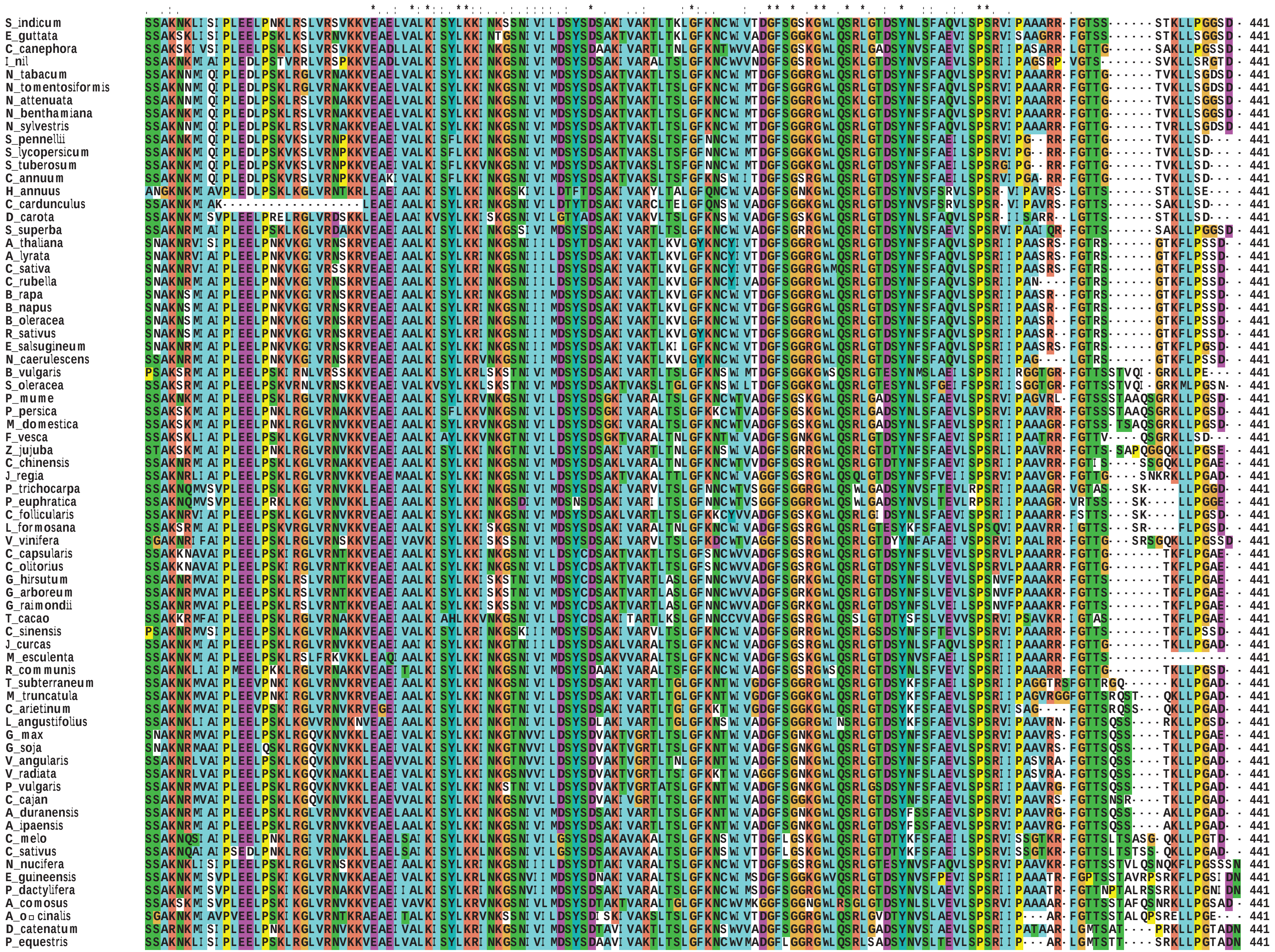

Supplementary Data S2: Sequence alignment of CAS proteins from monocots

```
O_sativa        ------MAPLSVSAILAP--SPPPAQA---AARASPRRAPASAA----PVAAAISTALLALTP-------AAHAAAFSKEDVAGSVTKVVDTVDQVIGVGGKVAEQSAGVLKAPGEAAKP----ALPALKSAGEQALKLASPVVSGASKQ 124
O_brachyantha   ------MAPLPVSSILAP--SPPPP-----AAKVSPRRTPASAA----PVAAAVSAALLAVTP-------AARAAAFSKEDVAGSVTKVVDTVDQVIGVGGKVAEQSAGVLKTLGEAAKP----ALPVLKRAGEQALKLASPVVSGASKQ 122
A_tauschii      ----MAFTPTSASATLPP-SAP-PPPA---ATKGTPRRAPWNAAPL-AAAAASSTAALLAATP-------AARAAALSKEDVAGSFTKVVDTVDVVIGVGGKVAEQSFVVLRALGEAAKP----ALPVLQSAGEQALKLASPVVSDASRQ 129
B_distachyon    ------MPPMSVSAVLPPPPAPSPPAA---ITKSLPRRAPARLA----SAATASASALLAVTPP------AARAATFSTEDVAGSLTKVVDTVDQVIGVGGKVAELSVTVLRALGEAAKP----ALPVLQSAGEQALKLASPAVSDASRQ 127
H_vulgare       ----MAFTPTSVSATLPP-SAP-PPPA---ATKGTPRRAPWNAA----LMAASSTAALLAVTP-------AAHAAAFSKEEVAGSFTKVVDTVDVVIGVGGQVAEQSFVVLRALGEAAKP----ALPVLQSAGEQALKLASPVVSDASRQ 126
S_italica       ----MA--PMSASATLAPLAPPPP-KA---TARSPARRAPANAAS------IAGSAALLTLMP-------AAPAAALSKEDVADSLTKVVDTVDQAIGVGGKVAEQVSGVVKALGEAAKP----ALPVLKSATDEAVKLAAPVVSSASKQ 123
D_oligosanthes  ----MA--PMSVSATLAPLAPPPPPKA---AAR--ARRAPANAASLAASAAVAGSAALLTLTP-------AAPAAALSKEDVTGSLTKVVDTVDQAIGVGGKVAEQFAGVLKALGEAAKP----ALPVLKSATDEVVKLAAPVVSGASKQ 128
Z_mays          ----MAPVPVSVSATLAPPPAAPP-KT---TSRSWERRAP-ADAAFAAASSVAGSAALLTLTP-------AAPAAALSKEDVAGSLTKAVDTVSQAIDVGGKAAEQVAAVLKALGEAVKP----ALPVLKSASDEALKLAAPVVSAASKQ 130
S_bicolor       ----MA--PMSVPATLAPPAPPKA-SP---TARSSARRAPAADAASVAASSVAGSAALLTLTP-------AAPAAALSKEDVAGSLIKVVDTVDDVIGVGGKAAEQVAAVLKALGEAVQP----AFPVLKSASDEALKLAAPVVSGASKQ 129
C_semulata      ----MRTMAFRIAAVAAKPSPPTPPKS--ITPHKCRHVNPP--SLPMFPPASTTAVSLLALLSSPV--VSDAKAFSLPKEDLLSSLTTVEGALDQFEKVGSSVLDYTISLYRILADALKPTVEAAAPVVQQAGEGTLKFALPLVSEASKQ 140
H_ovalis        ----MAVRASAVTGRPSSTPSKSAPLA--PTRRTRRPSNLPPLLLQPSSTTAA--VSLLTLFCTPGSHFEEAKALTFPKEDIISSLSKVENTLDQVEKVSTNVLDYTVSAFKVLYGVLKPTIDVTAPMVQGASKEAVKAASPILSEASKQ 142
P_oceanica      ----MRSMAFRVAAVAAKPSSPPPPKSTTVTPISKSHQREPLSSLPLLPPASTTAVSLLALFSTPV--VGDAKAFSIPKEDLLSSLTKVEGAVDQVEKVGSSVLKYSMSVINVLFDALKPTVEAAAPVAQQAGEGALKIALPLVSKASKQ 144
L_minor         MRAMMTARAAAVT-RPPTPSLPSSPGS--SKVDVIARRLKAPMLPQPSATAAAASVSLLALFSAPAGSFIEAKAFSLPKEDILSSLTQVENTLEQAQAAGSQAVDFSRSVFDFITGVLKPAADVVLPAVTKAGEEAVKIASPVVSDASKQ 147
S_isoetifolium  ----MRTMAFRIAAVATKPSPPPPPKS--IARHKCRHVNPP--CLPLFPPASATAVSLLALFSTPV--ISDAKAFSLPKEDLLSSLTKVEGVLDQFEKVGSSVLDYTISIFKILADALKPTVEAAAPVVQQTGEGALNFALPLVSEASKQ 140

O_sativa        ATEALQGAGVDPAPVLSAAKTVADAAQQGTKVIDAAKPIASATVETIGSLGSADYVVVAGAAFLAYLLLPPAWSLLSFTLRGYKGDLTAAQALDMVTSQDYVLIDVRTEKDKAKTGVPQLPSNAKNKLISIPLEELPSKMKSMVRNAKQA 274
O_brachyantha   ATEALQGAGVDPAPVLSAAKT---AAQQGTKVIDAAKPIASATVETIVSWGSADYVVAAGAAFLAYLLLPPAWSLLSFTLRGYKGDLTAAQALDMVTSQDYVLIDVRTEKDKAKAGVPQLPSNAKNKLISIPLEELPSKTKSMVRNAKQA 269
A_tauschii      ATEALQGAGVDLARFQSAFKTVADAAQP---AIGAAKPIASETVQTIGSLEGTDYVVAAGAAFLAYLLLPPAWSLLSYGLRGYKGDLSPAQALDMVTSQGYLIIDVRSENDKAKAGVPQLPSNAKNKIIALPLEELPNKIKGMVRNAKRA 276
B_distachyon    ATAALQGAGVDPAPVLSAAKTFADAAQQGGKVVDAAKPVASAAVETIVSFGAADYVVAAGAALLAYLLLPPALSLVSYSLRGYKGDLSPAQVLDMVTSQGYLLVDVRSEKDKGKAGVPQLPSNAKNKLISLPLEELPKKIKDMVRNARRT 277
H_vulgare       ATEALQGAGVDLAPLQSAFKTFADAAQP---AIGAAKPIASETVQTIGSLEGTDYVVAAGAAFLAYLLLPPAWSLLSYGLRGYKGDLNPAQALDMVTSQGYLIIDVRSENDKGKAGVPQLPSNAKNKIISLPLEELPNKIKGMVRNAKRA 273
S_italica       ATEALQGAGVDPAPVLTAAKT---AAEQGTKVIDAAKPVASATVETITSLAPGDYVVAAGAAFLAYLLLPPVWSLVSFTLRGYKGELTPAQALDKVTTKDYVLIDVRTDKDKAKAGVPQLPSNAKNKLISVPLEELPSKLKGMVRSAKKA 270
D_oligosanthes  ATEALQGAGVDPAPVLSAAKT---AAEQSTKVIDAAKPVASATVETITSLAPEDIVVVAGAAFLAYLLLPPVWSLVSFGLRGYKGELTPAQALDKVTTQNYILIDVRTDKDKAKAGVPQLPSNAKNKLIYVPLEELPSKLKGMVRNAKKA 275
Z_mays          ATEALQGAGVDPAPVLSVAKT---AAEQSTKVIDAAKPVASAAVETITSLGPEDYVVAAGXAFLAYLLVPPVWSLVSSSLRGYKGDLTPAQALDKVTTQGYVLIDVRSEKDKAKAGLPQLPSNAKNKLVSVPLEDLPSKLKGMVRNAKKA 277
S_bicolor       ATEALQGAGVDPAPVLSVAKT---AAEQSTKVIDAAKPVASATVETITLGPEGYVVTAGAALLAYLLVPPVWSLVSFSLRGYKGDLTPAQALDKVTTQDYVLIDVRSDKDKAKAGVPQLPSNAKNQLVSVPLEDLPSKLKGMVRNAKKA 276
C_semulata      AQEALQGAGVDPIPVISAAKTVVDAARQSTQLIDEAKPIASATFESIVSNDPTVIAGSAGALFLAYLLLPPVWSVISFGLRGYKGKLSPAQTLDLVTTQNYLMDVRAEKDKTKAGIPILPSNAASKLISLPLEELPSKIKAIVRDPKKV 290
H_ovalis        AQGVLQDAGVDPRPVL---KTVAGVAEQSTKIIEGTKPVASSAFETLSS-DPSALLGTAGALFLAYLLLPAVWSGISFTFRGYKGDLSPAKALDLVCSESYVIVDIRSEKDKIKAGIPRLPSNAKNSIIYTPLEELPSKLKGLVRDVKKV 288
P_oceanica      AQEALQGAGVDPTPVTSAAKTVVDAAQQSTKVIEGAKPIASATFESIVSNDPAVIAGSAGALFLAYLLFPSVWSVISFGLRGYKGKLSPALTLDLVTTQNHLVIDIRAEKDKNKAGIPRLPSNAMSKLISLPLEELPSKIQALVRNPKKV 294
L_minor         AKEALEGAGVDPSPVISAIKTVSDAAKQTTSVIEVAKPIASSTMDTISSSDPLIIAASVGSLVLAYFLLPPVFSAISFSFRGYKGNLSPAQTLDLISSQQYLLIDIRSEKEKNKAGVPRLPSGATNRLISIPLEDLPSKIKNIVRDAKKA 297
S_isoetifolium  AQEALQGAGVDPIPVISAAKTAVDAAQQSTKLIEKAKPIASATFESIVSNDPTVIAGSAGALFLAYLLLPPVWSAISFGLRGYKGKLSPAQILDLVTAQNYLMDVRAEKDKTKAGIPRLPSNAASKLISLPLEELPSKIKAIVRDPKKV 290

O_sativa        EAEIAALKISYLKRIGKGSNVIIMDSYCDSSKIVAKTLNSVGFKNCWVMAGGFSGRKGWAQSRLGTDSYNLSVVEVVTPSRVIPAAAD-RLVTASSS-----ASRTTTSRKLLPGSVDG 387
O_brachyantha   EAEIAALKISYLKRIGKGSNVIIMDSYSDSSKIVARTLNGVGFKNCWVMAGGFSGRKGWAQSRLGTDSYNLSVVEVVTPSRVIPAAAD-RFVTASS------TSRATTSRKLLPGSVDG 381
A_tauschii      EAEIAALKISYLKRIGKGSNIVVMDSYGDNSKIVAKTLNSVGFKNCWVMAGGFSGRKGWAQSRLGTDSYNLSVVEVVKPSRVIPAAAE-RFVTVSST-----STPSRTSRKLLPGSVDS 389
B_distachyon    EAEIAALKISYLKRVGKGSNIVVMDSYCDNAKIVARTLNSVGFNNCWVMAGGFSGGKGWAQSRLGTDSYNLSVVEVVKPSRIIPASAG-RFVTASST-----STTSGTNRKLLPGSVDG 390
H_vulgare       EAEIAALKISYLKRIGKGSNIVVMDSYGDNSKIVAKTLNSVGFKNCWVMAGGFSGQKGWAQSRLGTDSYNLSVVEVIKPSRVIPAAAE-RFVTVSST-----STPSRTTRKLLPGSVDN 386
S_italica       EAEITALKISYLKKIGKGSNVIIMDSYNDISKTVAKTLNSVGFKNCWVMAGGFSGRKGWAQSRLGTDSFNLSVVEVVTPSRVIPAAAG-RIGTTAGRIGTTSSASRATSRKLLPGSAD- 387
D_oligosanthes  EAEITALKISYLKKIGKGSNVIIMDSYNDISKTMAKTLNSVGFKNCWVMTGGFSGRKGWAQSRLGTDSFNLSVVEVVTPSRVIPAGAA-RIGTTSSA-------SREPSRKLLPGSVD- 385
Z_mays          EAEIAALKISYLKKIGKGSNVIIMDSYSDVAKTVAKTLDSVGFKNCWVMAGGFSGRKGWAQSRLGTDSYNLSVVEVVTPSRVIPAVAGRRTGTTAARIGTASSASRATTRKLLPGGVD- 395
S_bicolor       EAEIAALKISYLKKIGKGSNVIIMDSYNDVSKTVAKTLNSVGFKNCWVMAGGFSGRKGWAQSRLGTDSYNLSVVEVVTPSRVIPAVAG-RTGTTSARI-----ASRATTRKLLPGSVD- 388
C_semulata      EAEILAFKISYLKRVDRGSNIVIMDAYCGTAKTIAKWLTAAGFKNVWIMAGGFSGGKGWLQCRLGTGSYNVTLAEVVSPSRVIPASVS-RFGTTNPI-------SRQPTRRLLPGSTD- 400
H_ovalis        EAEIVALKISYLKRVGKGSNIVILDSYSDSAKTVARLLNYIGFKNVWVVTDGFSGRKGWIQSRLSCDSYTTNFAEILSPSRVIPAASR--FGTTGS----------RGSQKFLTGKVDA 395
P_oceanica      EAEIVALKISYLKRVNRGSNIVIMDSYCDTAKTVAKWLTAVGFKNVWVMTGGFSGGRGWSQCRLGTDSYNVTLADVLLPSRVIPAAVA-RFGTTSSS-------SRQPTQRLLPRSVE- 404
L_minor         EAELAAVKISYLKRVNKGTNIVIMDSYSDVAKTVARTLTGLGFKNCWIMADGFSGSKGWLQSRLGADSYSTSLVEVLSPSRVIPAARS--FGTSSST---------RSSGKLLSGGVDV 405
S_isoetifolium  EAEIVALKISYLKRVDRGSKIVIMDSYCDTAKTIAKWLTAAGFKNVWIMAGGFSGGKGWSQCRLGTGSYNVTLAEVVSPSRVIPASVP-RFGSTKSS-------SRQPTRKLLSGSTD- 400
```

Supplementary Data S3: Sequence alignment of CAS proteins from gymnosperms

```
                 :*::*** **:.   *:..**:.  :*  .*::.*     .     ***     .     **:  *:*   *  **.:    *   :  **  ::**::  .***  *:***:*:*:**:**::**::.   .:*:***.**.::* :* :******:***:**.:***
P_sitchensis     --MASKAVGIYGLSG--TAAFLDKPFLS-SSVKPAFQVHHKAQGKQLPNAHRLRIVASA--KWDDNLR-KNSHYLPMLLSIFTFVGDAKAVTKEDIVTTLTKVEDAYQKLERATSSAYGVSKDFIEQALKTMKPAMDAAMPYIQKATDSA 150
P_glauca         --MASKTVGIYGLSG--TAAFLDKPFLS-SSVKPAFQVHHKAQGKQLPNAHSLRIVASA--KWDDNLR-KNSHYLPMLLSIFTFVGDAKAVTKEDIVTTLTKVEDAYQKLEGATSSAYGVSKDFIEQALKTMKPAMDAAMPYIQKATDSA 150
P_massoniana     --MASKAVGIYGLSG--TTGFLDKPFLS-GSVKPAFQTHHKNQGKQLSNAHSLRIVASA--KWDDNLD-KKSHYLPMLLSIFTFVGDANAVTKEDILTALTKVEDTYQKLQGVTSSAYGVSKEFIEQTIKNMKPAMDAAMPYIQKASDSA 150
T_wallichiana    MAASSRAVGISGLTAAKTAAVLDKTSLSGSSIQTAF--FNSFQGKHLCKPPCLRVIAAARGKWDDSVLQKNLHNLPIVLSILTSAGDAMAMTKEDIVTSLTKVEETYQQIEGATSTAYGFSKDVLEQILKAVKPAMDAAMPYLQKVSDSA 150
C_japonica       MAASSRAVGISGLTGAKTAALLDRSFISNSSIQTAA--FYRFQGKPSCRHPSLRLNATAKEKLDDSVPPKILYYLPTVFSILSSAGDAEAVTKEEIVTTLTQVEETYQQISGAASTAYGVSKDFLDQVLKIIKPAMDASMPYLQKATDSA 150

                 **:*:*** **:*:** **:.****.*.*:*.******:***********:**.**:**:*::*::*:***:*.  ..********:.***:*:*:**.*****:*:*.:**:*********** *****:*******:**.*****.*:.
P_sitchensis     VQIASPVASDVTQQAQKALESAGVDPKPVVEAAKTAAVVAGGAAEQTTKAIEGAKPVASATIDSLLSSDPIVLAGGAGALILLYLLAPSIFSTISFSVRGYKGDLSPAEALQLLTKQDYVMDVRTEKEKSKAGIPSVPRSAKNKLLSLA 300
P_glauca         VQIASPVASDVTQQAQKALESAGVDPKPVVEVAKTAAVVAGGAAEQTTKAIEGAKPVASATIDSLLSSDPIVLAGGAGALILLYLLAPSIFSTISFSVRGYKGDLSPAEALQLLTKQDYVMDARTEKEKSKAGIPSLPRSAKNKLLSLA 300
P_massoniana     VQIASPVASDVTQQAQKALESAGVDSKSVVEAAKTAAVVAGGAAEQTTKAIEGVKPIASATIDSLLSSDPIVLVGGAGALILLYLLVPSIFSTIAFSLRGYKGELTPAQALQLLTKQDYVMDIRTEKERSKAGIPSLPRSAKNKLFSLA 300
T_wallichiana    VQVASPVALDVTQKAQIALENAGVDTKPVLEAAKTAAVIAGGAAEQTTKAIEGAKPIASTTVDSIVSADPIILAEAAGALILLYLFVPSILSSIAFSFRGYKGDLTPAQALELLTKQDYVMDIRTEKEKSKAGIPSLPRPAKNKLVSIA 300
C_japonica       VQVATPVASDVSQKAQKALQDAGVDTKPVVEAAKTAAVIAGGAAEQTTKAFEGAKPVASSTIESLLSADPIVLAEAVGALILLYLIVPSILSSISFSFRGYKGDLTPPQALELLTKQDYVMDVRTEKEKSKAGIPSLPRPAKNKLVSIP 300

                 :*********:**:  *:*****::***********.**:*:********* **::**.***  .:*:*******.:************.:***:*:*.**:**:   *              :**:**.*:*:
P_sitchensis     VEEFPNKLRSLLRNSKKVEAEVAALKISYLKRLNKSSRVVIMDSYGDVAKTVARALTDLGFNSTWILTDGFSGGKGWLQSCLGTESYNASFAEILSPSRIIPAGRGRFGTTRSGSQATSRRLLAGGLDD 429
P_glauca         VEEFPNKLRSLLRNSKKVEAEVAALKISYLKRLNKNSRVVLMDSYGDVAKTVARALTGLGFNNTWILTDGFSGGKGWLQSCLGTESYNASFAEILSPSRIIPAGRGRFGTTRSGSQATSRRLLAGGLDD 429
P_massoniana     VEEFPNKLRSLLRNSKKVEAEVAALKISYLKRLNKNSRVVIMDSYGDVAKTVARALTGLGFSNAWVLTDGFSGGRGWLQSCLGTESYNASFAEILSPSRIIPAARGRFGTTSTGSQATSRRLLASGLDD 429
T_wallichiana    VEEFPNKLRSLLRKFKQVEAEVTSLKISYLKRLNKGSRIVIMDSYGDVAKIVAKSLTSLGFTHTWILTDGFSGNRGWLQSCLGTESYNTSFAQIFSSSRIIPTNSG------------TRRILAGGVDD 429
C_japonica       IEEFPNKLRSVLRKFKQVEAEVAALKISYLKRLNKGSRIVIMDSYGDVAKIVAKALTSLGFKNTWILTDGFSGGRGWLQSCLGTESYGTSFAQILSPSRVIPASSG------------TRRLLAGGVDE 429
```

Supplementary Data S4: Sequence alignment of CAS proteins from green algae

```
                     :      :    :   .   .  .  :.   *:       *:  :  ::    :.    *.      :* .*:*::::*****::*:.**  **:::::**::*::.                                   ::. : ::
V_carteri       MSPAQLMKSASRAAVRPARSAVRALAQQRTQAKSAVRKSLATGLLASSVLLQSQAARAAEADQLDTTVETVVGAVKATGEVVKAGISTIQTGVKILKEGYDVAAPIAKQAVDTVAPVVVEAAKKTVELAQPAIDAAAPAISNTVSDVLES 150
G_pectorale     -MATQLMKSARVSSVRPARSSVRALAQLRTEAKSAVPKSFAAGLLASSVLLQASAAR---ADELDTTVESVVGAVKATGEVVKAGISTVQTGVRILKDGYDAAAPIVKQAYDTVTPLVVEAAKSTVELAQPAIEAAAPAISNTVSDVLES 150
Ch_reinhardtii  ---MQLAN-APRLAARPARSAVKVFAQQRTQAKTAAPKTLAAGLVASSVLLQASAAR---ADELDSTVESVVGAVKATGEVVKAGITTLQSGVKILKDGYDVAAPYAREVVDAVGPVVVEAAKKTVELASPALEAAAPAISNTVSDVLES 150
T_socialis      --MAPVMMQSAQRAVRPTRSAVKAFAVQRTQPKTIVPKTFAAGLAAAGLLVQAGAARAAEADQLDSTVDTVVGAVKATGEAVKAGITTIQSGVKLLQES-------------------------------------------TVTDVLES 150
Ch_eustigma     ---MALQQPLRACSRSQLKIQATAQPIHQKLHKSAPRWREASTIGTSIACLSQAAPAVAETDGVDTTIEALVGAVKTAGDAVKIGISALDSGVQILKQGYEVAAPVLEQGVKTVFPVVEK-----------AIDAASPTLKAALPVLQDA 150

 .:: *     : .** : * .* ..*:*:*    .  :. .**:**.*:.*.*..:  : * .:     *  **:**::*:* .**::  :.*.**:*:*. ::** **:****.: :.*::::.**:: *****. *::* :**  ***
V_carteri       T-GVDLSAVTNAAGTVGEVAGTAVSAATPVARSLAEVLASSDPVTLGEYALGAVALYYLTPALF----GLFRGFAGELTAAVALDTVVNDASAVLIDIRSAREKEASGVPDVPGAASSKVLEVEFAALEDKKLRSQLKDPSFIEAQTTAL 300
G_pectorale     S-GVDLSAVTNAAGTVSDVAGTAVKAASPVATSVAGALAGSDPVTLGEYALGAVALYYLLPAFF----GLFRGYAGDVTSAVALDTVVNDASAVLIDIRPSREKEASGVPDVPGAAASKVLEVEFAALEDKKLRSQLKDPGFIEAQTTAL 300
Ch_reinhardtii  T-GVDLSAVSKTAGTVGDVAGSAVSVATPVARGLVDLLATSDPVTLGEFGLGAVALYYLSPLFF----GAFRGYAGDMTSAVALDTVVNDASSVLIDIRAIREKEASGVPDVPGAASSKLLEVEFAALEDKKLRSQLKNPQDIEAQTTAL 300
T_socialis      A-GVDFSSVSEAAGTVSDVAESAYSAASPVAQGLAEALSASDPLTLAEFGLGAVALYYVAPAVL----GLFRGFAGELTAAVALDTVAQDASAVLIDIRPAREKEASGVPDVPGASASKVLEVEFAALEDKKLRSQLKDPSFIEAQTTAL 300
Ch_eustigma     EKAIEGSIKGAALDTVLDSASKAATVASPLASKAATFIGSNDPITLGEYALAAFVFLNVAPPLLGLIAGLTRGYAGEITAAQVLDALS-EGSCVLVDVRSEKDKENSGIPDVPSSVSNKIIDFDFATIVDKKLRGALRDPNGLEATFTAL 300

**::*:*:. .:*:*****:*  *. **:***:**:.:.: * ****** **:******** :*:  :* : .:*  * : : : ****::
V_carteri       QIASLRRIGTSSKVILLDRYGASAEAVARELAKKGYSRVFIVAGGFDGRAGWIQSKLQIKPYTATNLTFAAPAFGARTGTTSTRRLPAPRA- 392
G_pectorale     QIAALRRIGSGSKVILLDRYGPQAEAVAKELAKKGYGKVYVVSGGFDGRAGWIQSKLQIKPYTATNVTFAAPAFG-RTGTTSTRRLPAPRA- 392
Ch_reinhardtii  QIASLRRINSGTKVILLDRYGPLAEAVARELAKKGYGKVYVVVGGFDGRNGWIQSKLQIKPYTATALTFSAPAFG-RTGTTSTRRLPAPRS- 392
T_socialis      QIASLRRIGTGSKVILLDRYGGVAEQVARELAKKGYSRTYIVTGGFDGRAGWIQSKLQIKPYTATNLTFSAPAFG-RTGTTSTRRLPAPRAL 392
Ch_eustigma     QISALKRVNGSTKIILLDRFGSDARAVAKELARKGFGKVFTVQGGFDGRNGWVQSKLQIKPAAASSPAFMSFPLG-TTRSGTRKALPAPKA- 392
```

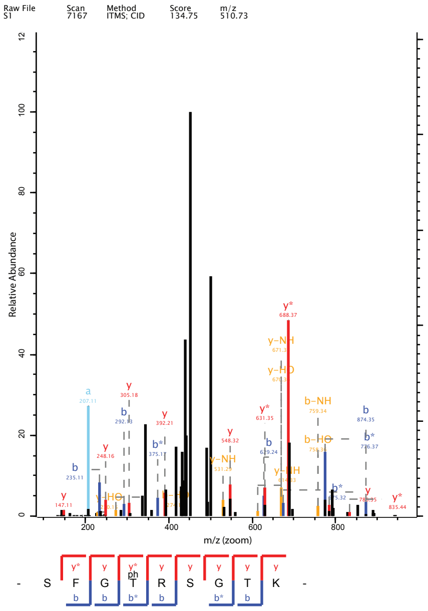

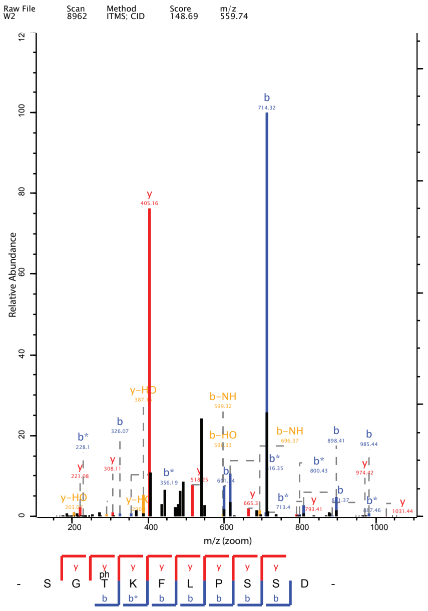

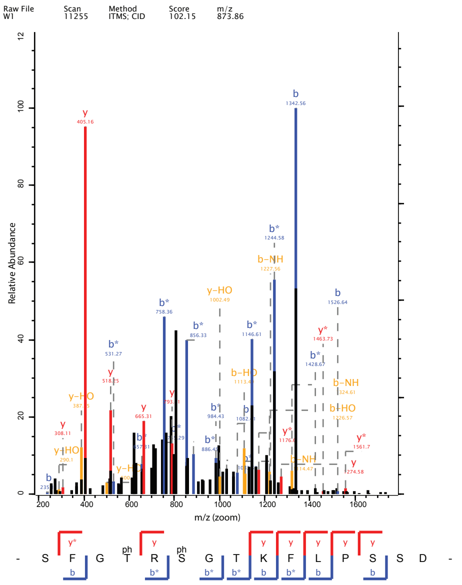

Supplementary Data S5:
MS/MS fragmentation spectra for the three phospho-peptides identified in the CAS protein. Positions of the phosphorylated residues are indicated.
