# Supplementary material for: The High Light Response in Arabidopsis Requires the Calcium Sensor Protein CAS, a Target of STN7- and STN8-Mediated Phosphorylation
Source: Front Plant Sci. 2019 Jul 30;10:974. doi: 10.3389/fpls.2019.00974 (PMC6682602; doi:10.3389/fpls.2019.00974)
Supplement: Supplementary file 2 [file Data_Sheet_2.PDF]

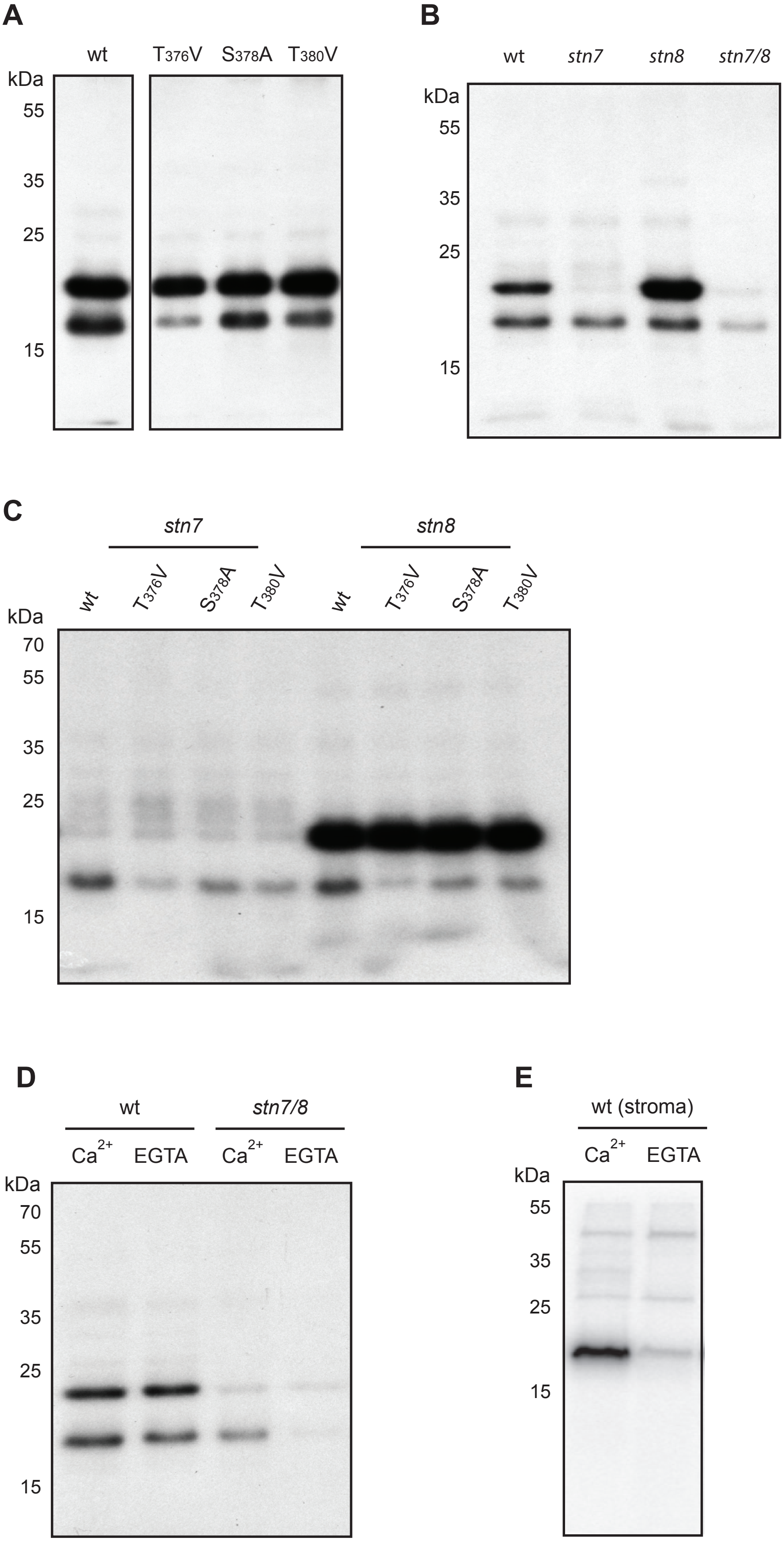

Supplementary Figure S1. (A - E) Full size images for the kinase assays shown in Figure 2A-E.

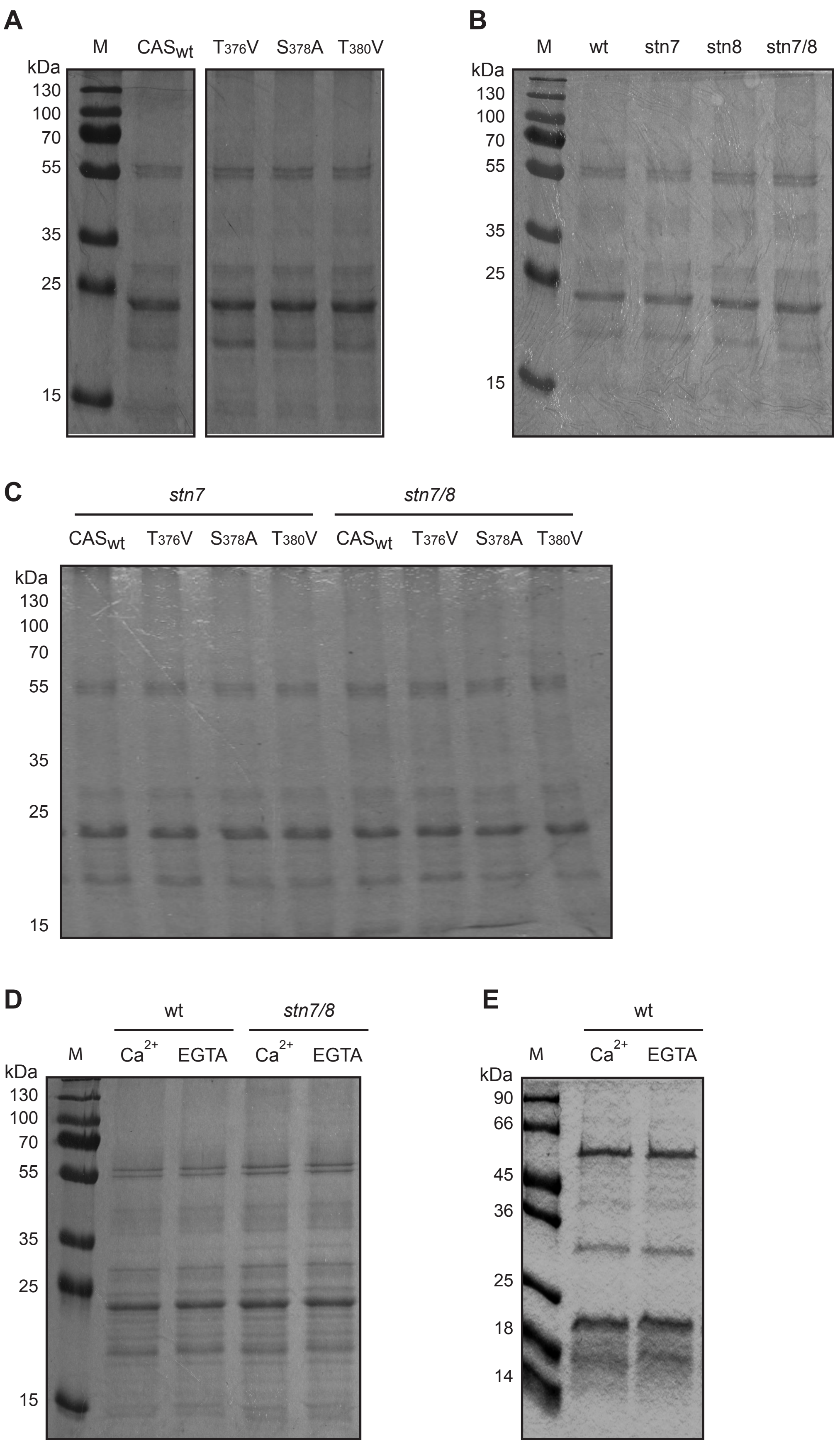

Supplementary Figure S2. Coomassie stained gels confirming equal loading of all samples analysed in Figure 2A-C **(A-C)** and Figure 5A and B **(D and E)**.

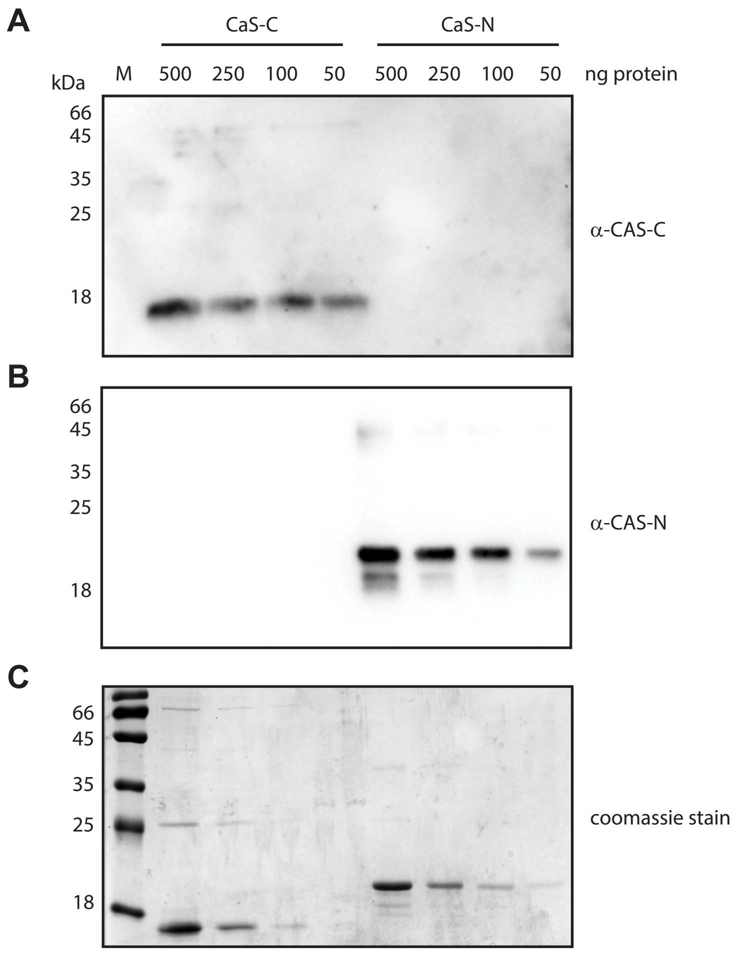

Supplementary Figure S3. Recombinant CAS-C (AAs 34-147) and CAS-N (AAs 216-387) fragments were tested with either **(A)** α-CAS-C or **(B)** α-CAS-N antiserum to show that the antibodies raised against the individual domains of CAS show no cross-reactivity. **(C)** Coomassie stained gel confirming equal loading of both recombinant proteins.

A

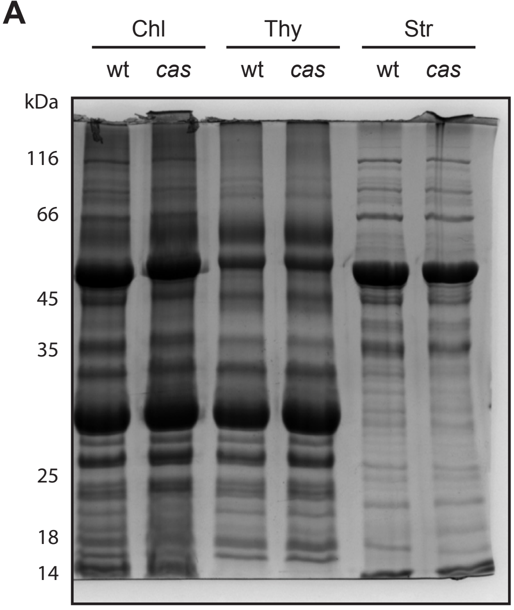

B

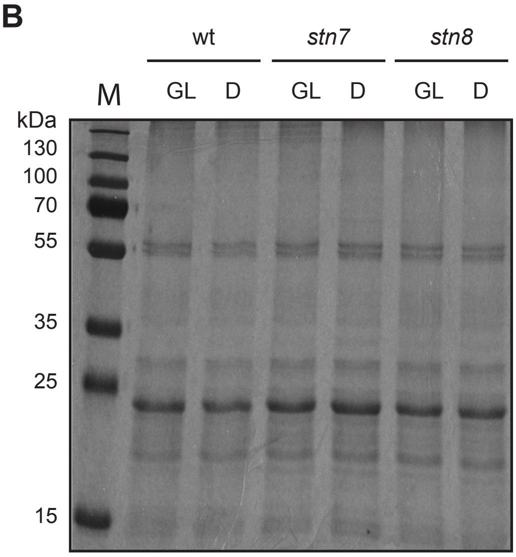

Supplementary Figure S4. Coomassie stained gel confirming equal loading of fractions analysed in **(A)** Figure 3C and **(B)** Figure 4 A.

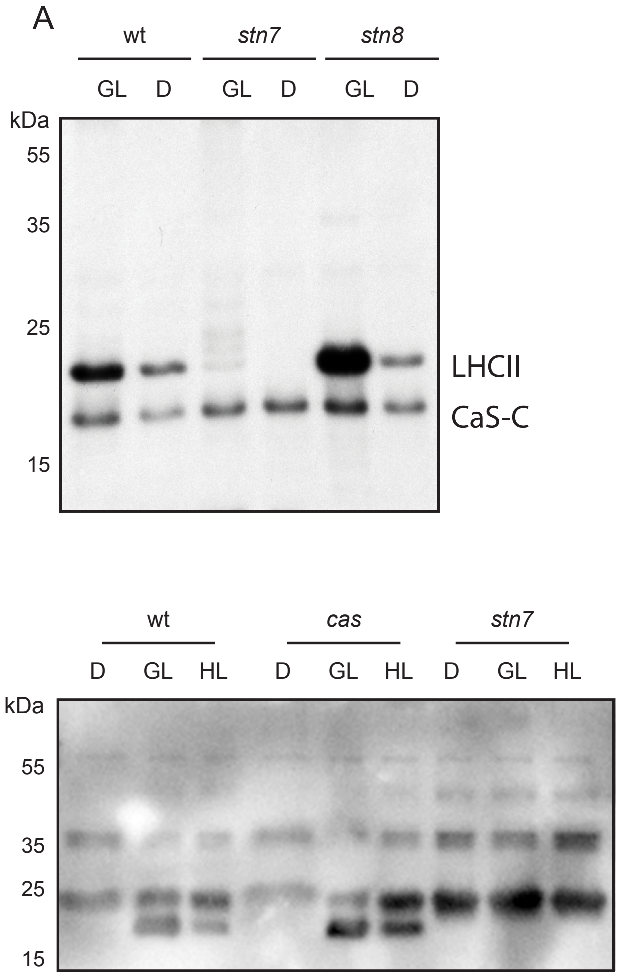

Supplementary Figure S5. **(A)** Full size image of the kinase assay shown in Figure 4A. **(B)** Full size image of the α-Thr Blots shown as inlays in Figure 4B.

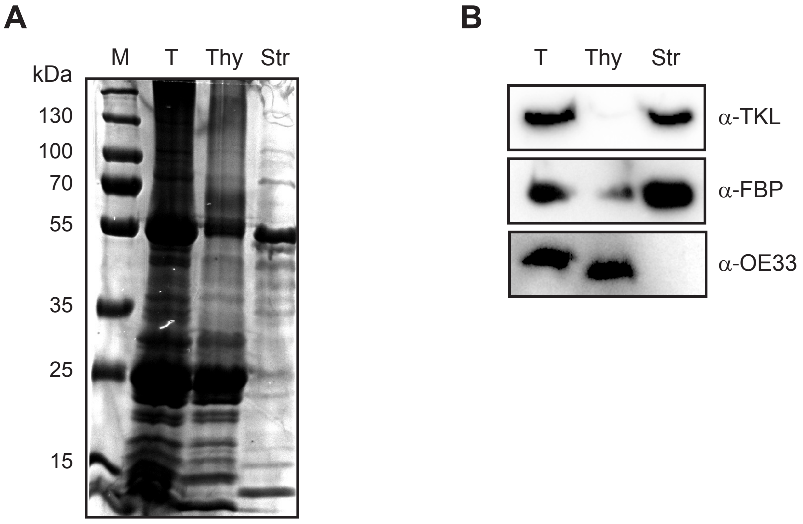

Supplementary Figure S6. Purity of the chloroplast subfractions used in kinase experiments was analysed by **(A)** Coomassie staining of proteins separated on SDS-PAGE and **(B)** Western Blot analysis using antibodies against transketolase (TKL), Fructose 1,6-bisphosphatase and 33 kDa subunit of the oxygen evolving system (OE33). T - total protein, Thy - thylakoid membrane fraction, Str - stroma.
