# Supplementary material for: The High Light Response in Arabidopsis Requires the Calcium Sensor Protein CAS, a Target of STN7- and STN8-Mediated Phosphorylation
Source: Front Plant Sci. 2019 Jul 30;10:974. doi: 10.3389/fpls.2019.00974 (PMC6682602; doi:10.3389/fpls.2019.00974)
Supplement: Supplementary file 3 [file Data_Sheet_3.PDF]

Supplementary Table S1. List of oligonucleotides used in this work

Primer Name	Sequence	Purpose
CAS_cDNA_fw	ATGGCTATGGCGGAAATGGC	Amplification of full length CaS CDNA
CAS_cDNA_rv	TCAGTCGGAGCTAGGAAGG	Amplification of full length CaS CDNA
CAS_fl+tp_ApaI_F	CCTGGGCCCATGGCTATGGCG GAAATGGC	Cloning of CaS-YFP fusion construct in pBIN (also for saGFP)
CAS_fl+tp_NotI_R	CTTGCGGCCGCCGTCGGAGCT AGGAAG	Cloning of CaS-YFP fusion construct in pBIN (also for saGFP)
CAS_fl-tp_ApaI_F	CCTGGGCCCGTTTCACTTCCA ACATCAACTTC	Cloning of CaS-YFP fusion construct in pBIN
RBCS-1A_Fw	GATGGGCCCATGGCTTCCTCT ATGCTCTCTTCCG	Cloning of RBCS-1A in pBIN-saGFP
RBCS-1A_Rev	TACGCGGCCGCCACCGGTGAA GCTTGG	Cloning of RBCS-1A in pBIN-saGFP
ntCAS_SapI_fw	GGTGGTTGCTCTTCCAACGTT TCACTTCCAACATC	Cloning of CaS-NT in pTWIN1
ntCAS_PstI_rv	GGTGGTCTGCAGTTACGTATC CATGGTCGATG	Cloning of CaS-NT in pTWIN1
CaS_T376V_F	GCTTTGGCGTTAGGTCCGGAA CCAAGTTCC	Site directed mutagenesis T375V
CaS_T376V_R	CGGACCTAACGCCAAAGCTTC TCGAAGCTG	Site directed mutagenesis T375V
CaS_S378A_F	GGCACTAGGGCCGGAACCAA GTTCCTTC	Site directed mutagenesis S378A
CaS_S378A_R	GAAGGAACTTGGTTCCGGCCC TAGTGCC	Site directed mutagenesis S378A
CaS_T380V_F	GGCACTAGGTCCGGAGTCAA GTTCCTTC	Site directed mutagenesis T380V
CaS_T380V_R	GAAGGAACTTGACTCCGGACC TAGTGCC	Site directed mutagenesis T380V

Supplementary Table S2. List of expression constructs used in this work

Construct	AGI Code	Vector	Reference
CaS-C	AT5G23060 (aa 216–387)	pTWIN1	Stael et al. 2011
CaS-C_{T350V}	AT5G23060 (aa 216–387)	pTWIN1	this study
CaS-C_{S373A}	AT5G23060 (aa 216–387)	pTWIN1	this study
CaS-C_{T376V}	AT5G23060 (aa 216–387)	pTWIN1	this study
CaS-C_{S378A}	AT5G23060 (aa 216–387)	pTWIN1	this study
CaS-C_{T380V}	AT5G23060 (aa 216–387)	pTWIN1	this study
CaS-N	AT5G23060 (aa 34–147)	pTWIN1	this study
CaS-YFP	AT5G23060 (aa 1–387)	pBIN-YFP	this study
ΔTP-CaS-YFP	AT5G23060 (aa 34–387)	pBIN-YFP	this study
saCaS-GFP_{1-10}	AT5G23060 (aa 1–387)	pBIN-$saGFP_{1-10C}$	this study
saCaS-GFP_{11}	AT5G23060 (aa 1 – 387)	pBIN- $saGFP_{11C}$	this study
saSSU-GFP_{1-10}	AT1G67090 (aa 1 – 181)	pBIN-$saGFP_{1-10C}$	this study
saSSU-GFP_{11}	AT1G67090 (aa 1 – 181)	pBIN- $saGFP_{11C}$	this study

Supplementary Table S3. List of CaS orthologs used in the phylogenetic analysis

Accession number	Species	Order	Division/Clade	Group
XP_011078735.1	*Sesamum indicum*	*Lamiales*	Angiosperms	Dicots
XP_012836576.1	*Erythranthe guttata*	*Lamiales*	Angiosperms	Dicots
CDO98499.1	*Coffea canephora*	*Gentianales*	Angiosperms	Dicots
XP_019167034.1	*Ipomoea nil*	*Solanales*	Angiosperms	Dicots
XP_016458050.1	*Nicotiana tabacum*	*Solanales*	Angiosperms	Dicots
XP_009607865.1	*Nicotiana tomentosiformis*	*Solanales*	Angiosperms	Dicots
XP_009762078.1	*Nicotiana sylvestris*	*Solanales*	Angiosperms	Dicots
XP_019247644.1	*Nicotiana attenuata*	*Solanales*	Angiosperms	Dicots
BAM66423.1	*Nicotiana benthamiana*	*Solanales*	Angiosperms	Dicots
XP_015070026.1	*Solanum pennellii*	*Solanales*	Angiosperms	Dicots
XP_004236097.1	*Solanum lycopersicum*	*Solanales*	Angiosperms	Dicots
XP_006345067.1	*Solanum tuberosum*	*Solanales*	Angiosperms	Dicots
XP_016556216.1	*Capsicum annuum*	*Solanales*	Angiosperms	Dicots
OTF90663.1	*Helianthus annuus*	*Asterales*	Angiosperms	Dicots
KVI06483.1	*Cynara cardunculus*	*Asterales*	Angiosperms	Dicots
XP_017245888.1	*Daucus carota*	*Apiales*	Angiosperms	Dicots
FA51419.1	*Schima superba*	*Ericales*	Angiosperms	Dicots
Q9FN48	*Arabidopsis thaliana*	*Brassicales*	Angiosperms	Dicots
XP_002874110	*Arabidopsis lyrata*	*Brassicales*	Angiosperms	Dicots
XP_010497107.1	*Camelina sativa*	*Brassicales*	Angiosperms	Dicots
XP_006287921.1	*Capsella rubella*	*Brassicales*	Angiosperms	Dicots
XP_009136394.1	*Brassica rapa*	*Brassicales*	Angiosperms	Dicots
XP_013736900.1	*Brassica napus*	*Brassicales*	Angiosperms	Dicots
XP_013599226.1	*Brassica oleracea*	*Brassicales*	Angiosperms	Dicots
XP_018478757.1	*Raphanus sativus*	*Brassicales*	Angiosperms	Dicots
XP_006394572.1	*Eutrema salsugineum*	*Brassicales*	Angiosperms	Dicots
JAU51671.1	*Noccaea caerulescens*	*Capparales*	Angiosperms	Dicots
XP_010695780.1	*Beta vulgaris*	*Caryophyllales*	Angiosperms	Dicots
KNA14182.1	*Spinacia oleracea*	*Caryophyllales*	Angiosperms	Dicots
ACU11587.1	*Liquidambar formosana*	*Saxifragales*	Angiosperms	Dicots
XP_010663515.1	*Vitis vinifera*	*Vitales*	Angiosperms	Dicots
XP_006374339.1	*Populus trichocarpa*	*Malpighiales*	Angiosperms	Dicots
XP_011012408.1	*Populus euphratica*	*Malpighiales*	Angiosperms	Dicots
GAV63690.1	*Cephalotus follicularis*	*Oxalidales*	Angiosperms	Dicots
XP_008244069.1	*Prunus mume*	*Rosales*	Angiosperms	Dicots
XP_007209216.1	*Prunus persica*	*Rosales*	Angiosperms	Dicots
XP_008360891.1	*Malus domestica*	*Rosales*	Angiosperms	Dicots
XP_004299254.1	*Fragaria vesca*	*Rosales*	Angiosperms	Dicots
XP_015883197.1	*Ziziphus jujuba*	*Rosales*	Angiosperms	Dicots
AEP13979.1	*Castanopsis chinensis*	*Fagales*	Angiosperms	Dicots
XP_018858081.1	*Juglans regia*	*Fagales*	Angiosperms	Dicots
OMO60951.1	*Corchorus capsularis*	*Malvales*	Angiosperms	Dicots
OMO63928.1	*Corchorus olitorius*	*Malvales*	Angiosperms	Dicots
XP_016712995.1	*Gossypium hirsutum*	*Malvales*	Angiosperms	Dicots
XP_017629010.1	*Gossypium arboreum*	*Malvales*	Angiosperms	Dicots
XP_012468788.1	*Gossypium raimondii*	*Malvales*	Angiosperms	Dicots
EOY22243.1	*Theobroma cacao*	*Malvales*	Angiosperms	Dicots
XP_006494243.1	*Citrus sinensis*	*Sapindales*	Angiosperms	Dicots
XP_012092927.1	*Jatropha curcas*	*Malpighiales*	Angiosperms	Dicots
OAY59312.1	*Manihot esculenta*	*Malpighiales*	Angiosperms	Dicots
XP_002512005.1	*Ricinus communis*	*Malpighiales*	Angiosperms	Dicots
GAU19019.1	*Trifolium subterraneum*	*Fabales*	Angiosperms	Dicots
XP_003609151.2	*Medicago truncatula*	*Fabales*	Angiosperms	Dicots

XP_004508601.1	*Cicer arietinum*	*Fabales*	Angiosperms	Dicots
XP_019463659.1	*Lupinus angustifolius*	*Fabales*	Angiosperms	Dicots
NP_001239903.1	*Glycine max*	*Fabales*	Angiosperms	Dicots
KHN15173.1	*Glycine soja*	*Fabales*	Angiosperms	Dicots
XP_017423515.1	*Vigna angularis*	*Fabales*	Angiosperms	Dicots
XP_014505979.1	*Vigna radiata*	*Fabales*	Angiosperms	Dicots
XP_007155178.1	*Phaseolus vulgaris*	*Fabales*	Angiosperms	Dicots
XP_020226796.1	*Cajanus cajan*	*Fabales*	Angiosperms	Dicots
XP_015944026.1	*Arachis duranensis*	*Fabales*	Angiosperms	Dicots
XP_016193993.1	*Arachis ipaensis*	*Fabales*	Angiosperms	Dicots
XP_008455203.1	*Cucumis melo*	*Cucurbitales*	Angiosperms	Dicots
XP_004137457.1	*Cucumis sativus*	*Cucurbitales*	Angiosperms	Dicots
XP_010262208.1	*Nelumbo nucifera*	*Proteales*	Angiosperms	Dicots
XP_010913586.1	*Elaeis guineensis*	*Arecales*	Angiosperms	Dicots
XP_008782048.1	*Phoenix dactylifera*	*Arecales*	Angiosperms	Dicots
XP_020090059.1	*Ananas comosus*	*Poales*	Angiosperms	Dicots
KZV16003.1	*Boea hygrometrica*	*Lamiales*	Angiosperms	Dicots
XP_015622662.1	*Oryza sativa*	*Poales*	Angiosperms	Monocots
XP_015688987	*Oryza brachyantha*	*Poales*	Angiosperms	Monocots
XP_003570449	*Brachypodium distachyon*	*Poales*	Angiosperms	Monocots
TraesCS6B01G320900.1	*Triticum aestivum*	*Poales*	Angiosperms	Monocots
BAJ86075	*Hordeum vulgare*	*Poales*	Angiosperms	Monocots
XP_020155630	*Aegilops tauschii*	*Poales*	Angiosperms	Monocots
XP_004953792	*Setaria italica*	*Poales*	Angiosperms	Monocots
OEL36345	*Dichanthelium oligosanthes*	*Poales*	Angiosperms	Monocots
NP_001150975.1	*Zea mays*	*Poales*	Angiosperms	Monocots
XP_002452613.1	*Sorghum bicolor*	*Poales*	Angiosperms	Monocots
lcl|CS015345	*Cymodocea semulata*	*Alismatales*	Angiosperms	Monocots
lcl|SI042933	*Syringodium isoetifolium*	*Alismatales*	Angiosperms	Monocots
lcl|PO069894	*Posidonia oceanica*	*Alismatales*	Angiosperms	Monocots
lcl|HO077784	*Halophila ovalis*	*Alismatales*	Angiosperms	Monocots
lcl|LM102276	*Lemna minor*	*Alismatales*	Angiosperms	Monocots
XP_006858246.1	*Amborella trichopoda*	*Amborellales*	Angiosperms	Monocots
ABK25223	*Picea sitchensis*	*Pinales*	Gymnosperms	
BT109530	*Picea glauca*	*Pinales*	Gymnosperms	
AFA51418	*Pinus massoniana*	*Pinales*	Gymnosperms	
AK406769.1	*Cryptomeria japonica*	*Pinales*	Gymnosperms	
AEQ59234	*Taxus wallichiana*	*Pinales*	Gymnosperms	
KXZ43342.1	*Gonium pectorale*	*Chlamydomonales*	Chlorophyta	
XP_002947936.1	*Volvox carteri*	*Chlamydomonales*	Chlorophyta	
GAX74748.1	*Chlamydomonas eustigma*	*Chlamydomonales*	Chlorophyta	
XP_001702364.1	*Chlamydomonas reinhardtii*	*Chlamydomonales*	Chlorophyta	
PNH01519.1	*Tetrabaena socialis*	*Chlamydomonales*	Chlorophyta	
XM_024516323	*Physcomitrella patens*	*Funariales*	Bryophyta	
OAE30096	*Marchantia polymorpha*	*Marchantiales*	Marchatiophyta	
XP_002972782.1	*Selaginella boe*	*Selaginellales*	Lycopodiophyta	
ABD64881.1	*Pteris vittata*	*Polypodiales*	Pteridophyta	
